# Protein Phosphatase 1 Recruitment by Rif1 Regulates DNA Replication Origin Firing by Counteracting DDK Activity

**DOI:** 10.1016/j.celrep.2014.02.019

**Published:** 2014-03-20

**Authors:** Anoushka Davé, Carol Cooley, Mansi Garg, Alessandro Bianchi

**Affiliations:** 1 Genome Damage and Stability Centre, School of Life Sciences, University of Sussex, Brighton BN1 9RQ, UK

## Abstract

The firing of eukaryotic origins of DNA replication requires CDK and DDK kinase activities. DDK, in particular, is involved in setting the temporal program of origin activation, a conserved feature of eukaryotes. Rif1, originally identified as a telomeric protein, was recently implicated in specifying replication timing in yeast and mammals. We show that this function of Rif1 depends on its interaction with PP1 phosphatases. Mutations of two PP1 docking motifs in Rif1 lead to early replication of telomeres in budding yeast and misregulation of origin firing in fission yeast. Several lines of evidence indicate that Rif1/PP1 counteract DDK activity on the replicative MCM helicase. Our data suggest that the PP1/Rif1 interaction is downregulated by the phosphorylation of Rif1, most likely by CDK/DDK. These findings elucidate the mechanism of action of Rif1 in the control of DNA replication and demonstrate a role of PP1 phosphatases in the regulation of origin firing.

## Highlights

• Rif1 recruits protein phosphatase 1 to telomeres and DNA replication origins
• PP1 docking motifs mediate the effect of Rif1 on DNA replication timing
• The PP1 recruitment activity of Rif1 counteracts DDK action on Mcm4
• Mutations in putative CDK/DDK sites near the PP1 motifs in Rif1 affect PP1 recruitment

## Graphical Abstract

**Figure:**
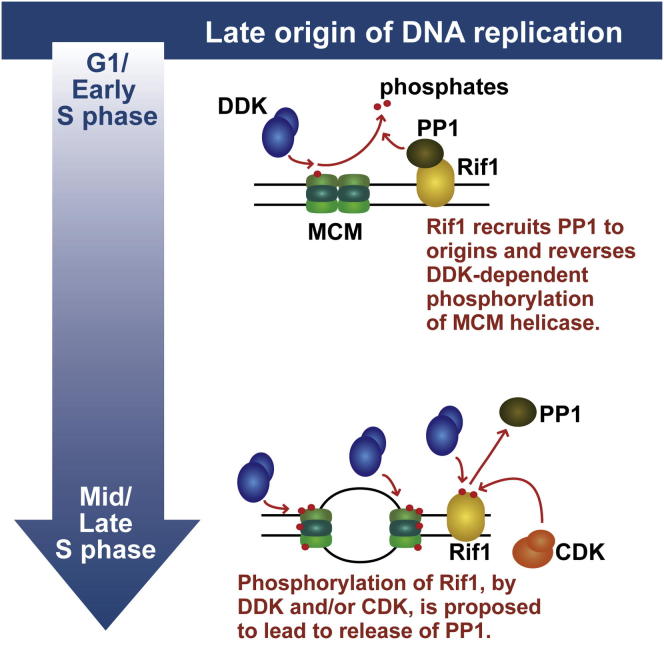

## Teaser

The eukaryotic genome is replicated according to a strict temporal program. Here, Bianchi and colleagues find that Rif1, a master regulator of the DNA replication program in yeast and mammals, exerts its effect on DNA replication origins by recruiting protein phosphatase 1 (PP1) to chromosomes. Rif1/PP1 counteracts the positive action of the DDK kinase on the replicative kinase MCM. In a final twist, kinase action on Rif1 is proposed to eventually release PP1 and allow origin firing.

## Introduction

The replication of Eukaryotic genomes is a highly regulated process. DNA replication starts at defined positions in the genome, called origins, the activation of which is strictly confined to the S phase of the cell cycle (Labib, 2010). Binding of the heterohexameric MCM helicase to a DNA-bound origin recognition complex (ORC) constitutes a first step in the assembly of a functional origin complex, or prereplication complex (pre-RC). The pre-RC is then activated by the action of the cyclin- and Dbf4-dependent kinases (CDK and DDK, respectively) at the end of the G1 phase. The essential function of CDK in DNA replication is the phosphorylation of the Sld2 and Sld3 proteins (Tanaka et al., 2007, Zegerman and Diffley, 2007), whereas the main role of DDK appears to be the phosphorylation of the MCM helicase, particularly the Mcm4 subunit (Sheu and Stillman, 2010). MCM phosphorylation allows recruitment of Cdc45/Sld3 and the GINS complex, which immediately precede polymerase loading and replication start (Heller et al., 2011, Tanaka et al., 2011).

However, these events do not take place simultaneously at all origins at the outset of S phase but are strictly choreographed, with origins being activated in a defined sequence that is a characteristic of each genome (Aparicio, 2013, Yoshida et al., 2013). Thus, origins can be broadly classified into early and late firing ones, based on their time of activation and, as a consequence, on their ability to fire in the presence of the drug hydroxyurea (HU). Exposure to HU leads to nucleotide depletion and activation of the intra-S phase replication checkpoint with subsequent inhibition of late-origin firing (Zegerman and Diffley, 2010).

The execution of an ordered program of origin activation is a conserved feature of Eukaryotic chromosomes, suggesting that it has an important function in the preservation of the genome (Müller and Nieduszynski, 2012). It remains, however, largely unclear how this program is established. In principle, the task can be achieved by either actively promoting the activity of early origins or by inhibiting that of the late ones, or by a combination of the two (Yoshida et al., 2013). In budding yeast (*Saccharomyces cerevisiae*) and metazoans, early origins appear to selectively benefit from the action of a limited supply of some of the key factors necessary for origin activation, including Cdc45 and the DDK subunit Dbf4 (Collart et al., 2013, Mantiero et al., 2011, Tanaka et al., 2011). It is not known what allows preferential action of these factors at the early origins, and not at the later ones. Clustering of the origins in defined nuclear regions appears to play a role (Duan et al., 2010), as highlighted by a function for the forkhead transcription factors in promoting origin-origin interactions at early replicating regions of the budding yeast genome (Knott et al., 2012).

On the other hand, origin-repressing activities have also been described. An inhibitory function of chromatin on origin action is documented by the role of telomeres (Ferguson and Fangman, 1992), which are late-replicating in yeast, and of heterochromatin-inducing activities such as histone deacetylases (Knott et al., 2009, Vogelauer et al., 2002), in delaying origin firing. A correlation between the nuclear positioning of origins in G1 and their replication timing has been observed (Heun et al., 2001), but artificial tethering of an early origin to the nuclear periphery in yeast (Ebrahimi et al., 2010) or introduction of mutations affecting delocalization of telomeres from the nuclear periphery (Hiraga et al., 2006) were not sufficient to change the replication timing of these regions, suggesting that the role of nuclear positioning in determining replication timing is likely to be complex and affected by several factors. At telomeres, the Sir3 and Ku proteins have been shown to be required for the late replication of budding yeast subtelomeric regions, suggesting that heterochromatin plays an important role in delaying origin firing at chromosome ends (Stevenson and Gottschling, 1999, Lian et al., 2011, Cosgrove et al., 2002).

The Rif1 protein, originally identified on the basis of its ability to interact with budding yeast telomeric DNA binding protein Rap1 (Hardy et al., 1992), has been found also to be required for the late replication of budding yeast telomeres (Lian et al., 2011). Subsequent work has revealed that, in both fission yeast (*Schizosaccharomyces pombe*) and mammalian cells, Rif1 acts as a general regulator of the origin firing program genome-wide (Hayano et al., 2012, Yamazaki et al., 2012, Cornacchia et al., 2012). The current view is that Rif1 helps establish late-replicating domains and that removal of Rif1 has an indirect knockon effect on early origins. Although the effect of Rif1 on DNA replication is thought to be mediated by its association with chromatin, which in fission yeast only partly relies on its interaction with the telomeric DNA binding factor Taz1 (Tazumi et al., 2012), it remains unknown how Rif1 carries out its repressive action at origins.

## Results and Discussion

### Rif1 Interacts with Protein Phosphatase 1

**Figure 1:**
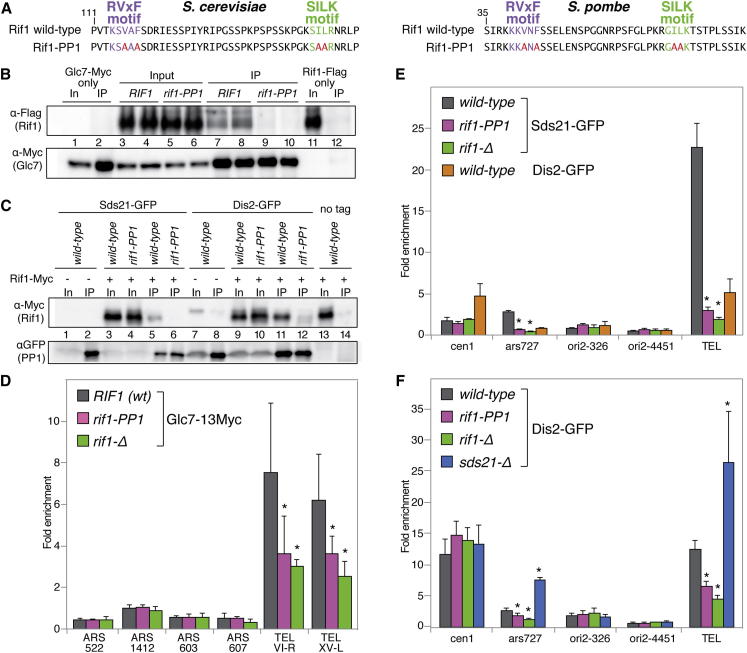
Rif1 Interacts with PP1 and Recruits It to Telomeres (A) Left: N-terminal sequence of ScRif1 spanning the putative PP1 docking motifs (top), which were mutated in the *rif1*-*PP1* allele (bottom). Right: N-terminal sequence of SpRif1 spanning the putative PP1 docking motifs (top), mutated in the *rif1-PP1* allele (bottom). (B) Protein extracts from budding yeast cells of the indicated genotypes were immunoprecipitated with anti-Myc and analyzed by western blotting against Flag (Rif1) and Myc (Glc7). (C) Protein extracts from fission yeast cells of the indicated genotypes were immunoprecipitated with anti-GFP and analyzed by western blotting against Myc (Rif1) and GFP (Sds21 and Dis2). (D) ChIP analysis of the association of ScGlc7 with the indicated chromosomal loci in the indicated strains, in exponentially growing asynchronous cultures. Fold enrichment was obtained by normalization against the PDI1 locus. SD values were derived from triplicates, and statistical significance was assessed by determining p values calculated from two-tailed t tests (in all cases, each mutant versus wild-type). ^∗^p < 0.05. (E) Association of N-terminally GFP-tagged SpSds21 and SpDis2 from exponentially growing asynchronous cultures at the indicated loci as determined by ChIP and quantified as fold enrichment over the *ars2004* locus. SDs and p values were calculated from four replicates. (F) ChIP analysis of SpDis2 chromatin binding as in (E); SDs and data are from four replicates. See Figure S1 for expression levels of mutant alleles.

Rif1 has two conserved putative protein phosphatase 1 (PP1) docking motifs (RVxF and SILK type) at its N terminus (Sreesankar et al., 2012). To test whether an interaction with PP1 is important for the role of Rif1 in the control of replication timing, we made an allele of budding yeast RIF1 (*Sc rif1-PP1* allele) carrying two substitutions in each of the conserved motifs (Figure 1A, left; see also Figure S1B). In budding yeast, a single member of the PP1 family is present, encoded by the essential *GLC7* gene, and therefore we set out to investigate whether Rif1 associates with Glc7. Indeed, Myc-tagged Glc7 was able to immunoprecipitate Flag-tagged Rif1 in cell extracts (Figure 1B, lanes 7 and 8), consistent with previous results (Breitkreutz et al., 2010). The amount of Rif1 in the immunoprecipitates was low, possibly as a reflection of low affinity of the interaction, or of differences in relative amounts of the two proteins, or, perhaps more likely, due to competition by other Glc7 binding partners. In any case, importantly, the interaction between the two proteins was not detected in the presence of the *rif1-PP1* mutations (Figure 1B, lanes 9 and 10). We then generated an analogous *rif1-PP1* allele in *S. pombe*, with the same changes in two of the conserved residues in each of the two PP1-interacting motifs (Figure 1A, right; also Figure S1F). In fission yeast, two PP1 family members are present, Dis2 and the less abundant Sds21 (Alvarez-Tabarés et al., 2007). Again, tagged versions of these PP1 proteins were able to immunoprecipitate epitope-tagged Rif1 (Figure 1C, lanes 5 and 11). In this yeast too, the presence of the *rif1-PP1* allele disrupted the interaction (Figure 1C, lanes 6 and 12). Although we, of course, cannot rule out that the interaction between Rif1 and PP1 proteins in either species is indirect, these results suggest that the PP1 docking motifs in Rif1 are functional and promote an interaction with the PP1 phosphatases.

### Rif1 Recruits PP1 to Telomeres and to a Late Origin of DNA Replication

The interaction between yeast Rif1 and PP1 raised the possibility that PP1 might be recruited to Rif1-bound chromosomal loci. In budding yeast, chromatin immunoprecipitation (ChIP) revealed robust association of Glc7 with telomeres, which bind Rif1 (Figure 1D). In addition, the binding of Glc7 at both telomeres tested, *VI-R* and *XV-L*, was greatly reduced in the absence of Rif1 and also in the presence of the *rif1-PP1* allele. These data demonstrate that the budding yeast PP1, Glc7, is associated with telomeres at least in part in a Rif1-dependent manner.

To address whether the role of the Rif1/PP1 interaction is restricted to telomeres, we turned to fission yeast where, as in mammals, Rif1 controls replication timing genome-wide. We tested the association of the two *S. pombe* PP1 homologs, Sds21 and Dis2, with a number of chromosomal loci, including a telomere-adjacent region common to the four telomeres of chromosomes I and II, and several origins of DNA replication: the early-firing origins *ori2-326* and *ars2004*, and the late-firing *ars727* and *ori2-4451*, in addition to the centromere of chromosome I (Figure 1E). Because levels of *ars2004* DNA in the immunoprecipitates were very low and independent of *rif1* allelic status (data not shown), we normalized all data to *ars2004*. This analysis revealed strong binding of Sds21 at telomeres that, as observed for Glc7 in *S. cerevisiae*, greatly diminished in the absence of Rif1 or in the presence of Rif1-PP1 (Figure 1E). Interestingly, telomere binding of Dis2 was lower compared to Sds21 (although the latter is less abundant within cells), whereas at *cen1* the situation was reversed and binding of Dis2 was higher compared to Sds21. These results suggest that Sds21 is the primary binding partner of SpRif1 at telomeres, whereas Dis2 might function primarily at centromeres. Importantly, we were able to detect Sds21 binding to one late origin of DNA replication, *ars727*, which previous work has shown to be bound by Rif1 (Figure 1E) (Hayano et al., 2012). Like at telomeres, the binding of Sds21 at *ars727* was strongly affected by mutation of *rif1*. We could not detect binding of Sds21 to the two early origins. However, we also failed to detect Sds21 at the late-firing Rif1-associated origin *ori2-4451*: it is possible that our PCR primers in this case are simply located in an area of low or absent Rif1 binding, which is not homogeneously distributed over late-firing regions (Hayano et al., 2012). Although it remains unclear how pervasively Sds21 (and possibly Dis2) associate with fission yeast origins genome-wide, our results establish that Rif1 recruits PP1 phosphatases to late-replicating telomeric regions in both budding and fission yeast, and to at least one nontelomeric late-firing origin in fission yeast, suggesting that PP1 recruitment is likely to take place at other Rif1-bound origins.

Although Sds21 and Dis2 localize to different cellular and nuclear compartments, there is a degree of overlap in both their localization and function. Dis2, unlike Sds21, is associated with centromeres (Figure 1E), and its absence leads to increased expression and redistribution of Sds21 to these sites, where it is not otherwise normally visualized (Alvarez-Tabarés et al., 2007). Although Sds21 seems to have the primary role in binding to telomeres and to *ars727*, the binding of Dis2 to these loci is also dependent on Rif1 (Figure 1F). Interestingly, in both instances, deletion of the *sds21*^*+*^ gene lead to an increase of about 2.5-fold in the association of Dis2. Thus, similarly to what was previously observed concerning the ability of Sds21 to replace Dis2 at centromeres in its absence, Dis2 increases its association at loci normally occupied by Sds21 in the absence of the latter. The binding of Dis2 at the centromere was instead unaffected either by mutations in Rif1 or Sds21.

### The PP1-Interacting Motifs of Rif1 Are Required to Establish the Replication Timing of Telomeric and Nontelomeric Loci

**Figure 2:**
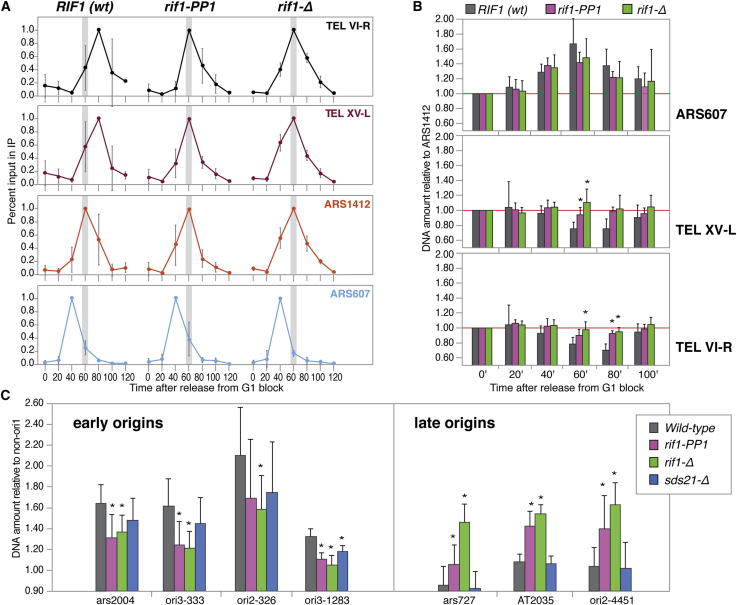
The PP1 Docking Motifs in Rif1 Are Required to Establish the Replication Timing of Budding Yeast Telomeres and Fission Yeast DNA Replication Origins (A) Analysis of the association of C-terminally Myc-tagged Pol2 with selected telomeres and origins in *RIF1* wild-type, *rif1-PP1*, and *rif1-Δ* budding yeast cells after synchronous release from G1 arrest. ARS607 (blue) and ARS1412 (orange) were used as markers of early and late S phase, respectively. To account for differences in efficiencies in the immunoprecipitations among different experiments, each profile for each amplicon was normalized against its highest peak. The data represent the average of three independent experiments for each strain. The significance of the change in the position of the telomere peak for each rif1 mutant against the wild-type was assessed by applying a Wilcoxon test (one sided; p < 0.024). (B) Analysis of the replication timing of *ARS607* and the *VI-R* and *XV-L* telomeres, in reference to *ARS1412*. DNA amounts for cells after release from G1 arrest were quantified by quantitative PCR (qPCR). For each of the three loci analyzed, normalization was first carried out against the *ARS1412* locus at the same time point, and subsequently against the G1 time point (0 min). A minimum of three experiments were averaged for the analysis. Two-tailed t tests were carried out for significance for each mutant against wild-type at the same time point (p < 0.05 is indicated by asterisks). See also Figure S2. (C) Replication efficiency of early and late origins in fission yeast, in wild-type, *rif1-*Δ, *rif1-PP1*, and *sds21-*Δ strains. Log-phase cultures were arrested in G2 at 36°C using the *cdc25-22* temperature-sensitive allele and then released into 25 mM hydroxyurea for 140 min. Genomic DNA was prepared for the G2 (0 min time point) and late S phase (140 min time point) cells and quantified by qPCR. The ratio of the amount of genomic DNA in late S phase to that in G2 was calculated for each locus. The *non-ori1* locus was used for normalization (Hayano et al., 2012). Two-tailed t test for each mutant against wild-type were performed from at least eight replicates. A p value < 0.05 was deemed significant and is indicated by an asterisk in the graph. SDs are indicated in all panels.

We have previously shown that the timing of association of Pol2 with yeast telomeres reflects their timing of replication and is dependent on the timing of firing of subtelomeric origins (Bianchi and Shore, 2007). In our experiments, Pol2 association with the early origin *ARS607* peaks at 40 min after release from G1 phase, in early S phase, whereas the late origin *ARS1412* peaks at 60 min (Figure 2A, bottom two panels). Pol2 association with telomeres normally peaks even later in S phase, at 80 min after release (Figure 2A, top two panels, left). Instead, cells carrying the *rif1-PP1* allele displayed a change in telomere Pol2 association, peaking at 60 min, concomitant with binding at *ARS1412* (Figure 2A, top two panels, middle): the extent of the change in Pol2 association is indistinguishable to the one observed in the absence of Rif1 (Figure 2A, top two panels, right), suggesting that the changes in telomere replication timing previously described at budding yeast telomeres in cells lacking Rif1 (Lian et al., 2011) are due to reduced Glc7 telomere binding.

To obtain further evidence that PP1 binding by Rif1 leads to changes in timing of DNA replication at budding yeast telomeres, we quantified the amount of genomic DNA present during S phase progression. Because at the *ARS1412* origin neither the association of Pol2 (Figure 2A) nor the replication timing (Lian et al., 2011) is affected by Rif1, we normalized the data at each individual time point against this locus and against the G1 time point (0 min). In this manner, a locus being replicated before *ARS1412* is predicted to show an increase over the baseline (i.e., to result in values higher than 1) in advance of *ARS1412* replication before returning to the baseline after completion of DNA replication at both loci. Indeed, this is what we observed for the early-firing origin *ARS607* for all strains examined (Figure 2B, top panel). Instead, a locus replicating after *ARS1412*, should show a dip below the baseline coincident with *ARS1412* replication: as expected, this was observed in wild-type cells for both telomeres *VI-R* and *XV-L* (Figure 2B, middle and bottom panels). In contrast, no dip was observed for either telomere in cells lacking *RIF1* or carrying the *rif1-PP1* allele (Figure 2B, middle and bottom panels), indicating that the replication of these telomeres occurs at the same time as *ARS1412* in the presence of the *rif1* mutations, in agreement with the Pol2 ChIP data. These results demonstrate that replication timing at budding yeast telomeres is advanced in cells where the ability of Rif1 to interact with Glc7^PP1^ has been compromised.

Loss of Rif1 can suppress defects in DNA replication in cells that are impaired for DDK function in budding yeast (see below). Although it would seem unlikely that this effect is solely due to the effect of Rif1 at telomeres, we have so far failed to observe binding of ScRif1 (data not shown) or Glc7 (Figure 1D) at origins. However, we have documented mild effects of Rif1 at one late nontelomeric origin, *ARS603*, which would be consistent with a more global role of Rif1 on origin firing (Figure S2).

To further test whether the role of Rif1/PP1 in affecting origin function is widespread in the genome or confined to telomeres, we turned to fission yeast and took advantage of a well-characterized set of early and late origins (Hayano et al., 2012). Cultures were synchronized in the G2 phase at 36°C with a *cdc25-22* temperature-sensitive allele and released into the cell cycle at 25°C in the presence of hydroxyurea, to suppress firing at late origins. DNA amounts after incubation in HU were normalized against a locus (*non-ori*) that is not replicated under these conditions (Hayano et al., 2012), and against the amount at G2 arrest, to provide a measure of the ability of the origins to fire in HU, and therefore of their timing of firing. For the early origins *ars2004*, *ori3-333*, *ori2-326*, *and ori3-1283*, we observed a similar decrease in DNA amounts in *rif1-Δ* and *rif1-PP1* strains, compared to wild-type (Figure 2C, left panel). Analysis of the late origins *ars727*, *AT2035*, and *ori2-4451* instead yielded an increase in DNA amounts in both *rif1-Δ* and *rif1-PP1* strains (Figure 2C, right panel), indicative of a shift to early firing for these late origins. These results demonstrate that, at several loci tested in fission yeast, impairment of the ability of Rif1 to interact with PP1 leads to a loss of control of the timing of origin firing that phenocopies the misregulation observed in the absence of Rif1. We did not observe significant differences in replication efficiencies in the *sds21-Δ* strains compared to wild-type, presumably due to compensatory effects from Dis2.

### PP1 Recruitment by Rif1 Affects DDK Action on the MCM Helicase

**Figure 3:**
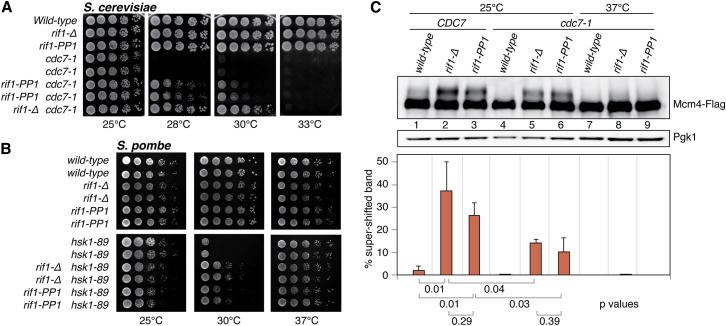
Recruitment of PP1 by Rif1 Counteracts DDK Activity on Mcm4 (A) Suppression of the temperature-sensitivity phenotype of the budding yeast *cdc7-1* allele by *rif1-PP1*. 5-fold serial dilutions of log-phase cultures of budding yeast strains of the indicated genotypes were plated onto YPAD media and incubated at temperatures ranging from 25°C to 33°C. Plates were imaged following 2 day incubations. (B) Suppression of the temperature sensitivity of the fission yeast *hsk1-89* allele by *rif1-PP1*. 10-fold serial dilutions of log-phase cultures of the indicated genotypes were spotted on rich medium and incubated for 4 days at 25°C or 3 days at 30°C and 37°C (the latter is a permissive temperature for *hsk1-89*). (C) Analysis of budding yeast Mcm4 phosphorylation. Budding yeast strains bearing Flag-tagged Mcm4 were arrested in the G1 phase with α-factor at 25°C or 37°C for 2 hr, as indicated. Western analysis of protein samples was performed with anti-Flag (top) and anti-Pgk1 (bottom). The phosphorylated fraction of the Mcm4 protein and the total Mcm4 signal were quantified using ImageQuant software and normalized to the loading control (Pgk1), and then the percentage of phosphorylation was calculated. The p values from two-tailed t tests are reported in the graph. At least three replicates were used for the analysis and SDs are indicated. See also Figure S3.

Loss of Rif1 restores viability of fission yeast cells lacking Hsk1, the catalytic subunit of DDK (Hayano et al., 2012). Similarly, we found that loss of Sc Rif1 partly suppresses the temperature sensitivity of an allele of the *hsk1*^*+*^ budding yeast ortholog, *cdc7-1* (Figure 3A). Remarkably, *rif1-PP1* was also able to partly suppress the temperature sensitivity of *cdc7-1*, although to a lesser extent than *rif1-Δ* (Figure 3A). Similarly to budding yeast, fission yeast *rif1-PP1* also restored growth to *hsk1-89* mutants, to an extent comparable to that conferred by *rif1-Δ* (Figure 3B). These results suggest that the Rif1-dependent recruitment of PP1 to replication origins might counteract DDK kinase activity at origins.

To test this idea directly, we assessed Mcm4 phosphorylation in cells carrying mutations in Rif1. Budding yeast Mcm4 has been shown to be a target of multiple phosphorylation events by CDK and DDK (Sheu and Stillman, 2006, Sheu and Stillman, 2010, Randell et al., 2010). Although phosphorylation of Mcm4 was not easily apparent in G1-arrested wild-type cells, a supershifted band was readily observable in cells lacking Rif1 (Figure 3C, compare lane 2 with lane 1). This phosphorylation was greatly diminished in the presence of the *cdc7-1* mutation at the permissive temperature and was undetectable at the nonpermissive one (Figure 3C, lanes 5 and 8). These results suggest that phosphorylation of Mcm4 is largely DDK dependent and that this phosphorylation is inhibited by the action of Rif1. Importantly, an increase in phosphorylation of Mcm4 was also observed, to a similar extent, in the presence of the *rif1-PP1* allele (Figure 3C, lanes 3 and 6), suggesting that PP1 activity recruited by Rif1 is responsible for reversal of DDK phosphorylation events.

We did not observe an ability of Rif1 to suppress various budding and fission yeast CDK mutants (Figure S3), but this could be conceivably due to the fact that CDK carries out multiple essential roles other than activation of origin firing. Indeed, the fact that absence of *rif1* suppresses an *hsk1*-null allele in *S. pombe* suggests that reversal of DDK-dependent phosphorylation is not the only function of Rif1/PP1, and that CDK-dependent phosphorylation events might be targeted as well.

### Rif1/PP1 Is Affected by Mutations at Putative CDK and DDK Phosphorylation Sites in the Rif1 N Terminus

**Figure 4:**
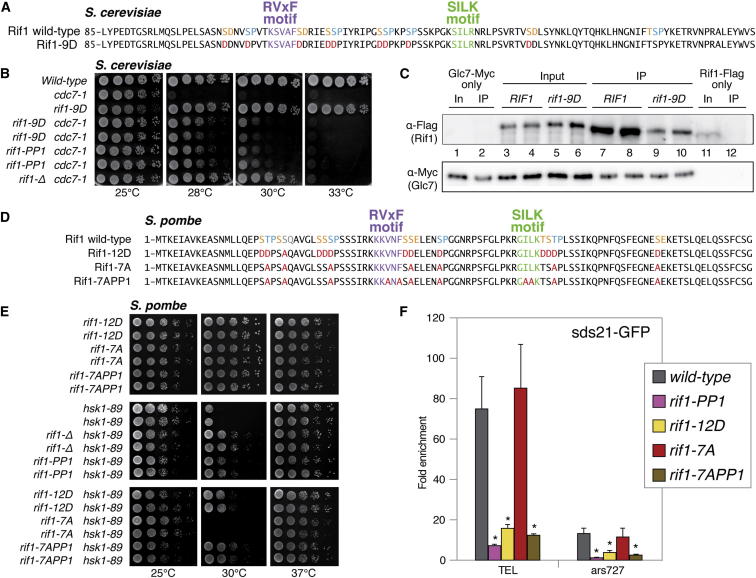
Rif1/PP1 Is Affected by Mutations at Putative CDK and DDK Phosphorylation Sites in the Rif1 N Terminus (A) N-terminal sequence of ScRif1 spanning the putative PP1 docking motifs (top). The RVxF- and SILK-type motifs are indicated in purple and green, respectively. Potential DDK sites are indicated in orange; putative CDK sites are indicated in blue (top). Phosphomimic changes to aspartic acid present in the *rif1*-*9D* allele are indicated in red (bottom). (B) Suppression of the temperature-sensitivity phenotype of the budding yeast *cdc7-1* allele by *rif1-9D*. 5-fold serial dilutions of log-phase cultures of budding yeast strains of the indicated genotypes were plated onto YPAD media and incubated at temperatures ranging from 25°C to 33°C. Plates were imaged following 2 day incubations. (C) Protein extracts from budding yeast cells of the indicated genotypes were immunoprecipitated with anti-Myc and analyzed by western blotting against Flag (Rif1) and Myc (Glc7). (D) N-terminal sequence of SpRif1 spanning the putative PP1 docking motifs (top). The RVxF- and SILK-type motifs, and the putative DDK and CDK sites are indicated as in (A). Changes to aspartic acid or alanine present in the *rif1*-*12D*, *rif1-7A*, and *rif1-7APP1* alleles are indicated in red (bottom). (E) Suppression of the temperature sensitivity of the fission yeast *hsk1-89* allele by various *rif1* alleles. Ten-fold serial dilutions of log-phase cultures of the indicated genotypes were spotted on rich medium and incubated for 4 days at 25°C or 3 days at 30°C and 37°C. (F) Association of N-terminally GFP-tagged SpSds21 from exponentially growing asynchronous cultures at the indicated loci as determined by ChIP and quantified as fold enrichment over the *ars2004* locus. SDs and p values for each mutant versus wild-type were calculated from three replicates. See Figure S1 for expression levels of mutant alleles.

The PP1 docking domains, in both Sc and Sp Rif1, are embedded in a conserved cluster of putative DDK and CDK sites (Figures 4A, 4D, S1B, and S1F), some of which are known to be phosphorylated (http://www.phosphopep.org). Because precedents exist for inhibition of PP1 binding upon phosphorylation of residues in the proximity of the docking motifs (Kim et al., 2010, Grallert et al., 2013), we considered the possibility that CDK- and DDK-dependent phosphorylation of Rif1 might inhibit PP1 binding, thus helping to enforce the chronological separation of origin firing throughout S phase, as activation of the pre-RC at Rif1-delayed origins would require the levels of the kinases to reach sufficiently high levels for inhibition of PP1 binding. To test this idea, we made mutations in several putative DDK and CDK sites found in the vicinity of the RVxF and SILK motifs in budding yeast Rif1 (Figure 4A). Specifically, we changed nine serine residues to aspartic acid to mimic constitutive phosphorylation at these sites, and then we assessed the ability of this mutant (*rif1-9D*) to suppress the temperature-sensitivity phenotype of the *cdc7-1* allele. In agreement with the idea that phosphorylation at these sites might suppress binding of Glc7 to Rif1, we found that the *rif1-9D* phosphomimic allele suppressed growth defects of *cdc7-1* cells to a similar extent as the *rif1-PP1* allele (Figure 4B). Coimmunoprecipitation analysis of this mutant supports this interpretation, because we found that the ability of Glc7 to interact with Rif1 was diminished by the presence of these amino acid substitutions (Figure 4C).

In order to test the potential role of phosphorylation within the N terminus of *S. pombe* Rif1 as well, we made similar phosphomimic substitutions in the protein (Figure 4D, *rif1-12D* allele). As observed in *S. cerevisiae* for *rif1-9D* and *cdc7-1*, the fission yeast allele showed an ability to improve the viability of *hsk1-89* cells (Figure 4E). Strikingly, a second allele where the possibility of CDK and DDK targeting these sites was eliminated by changing serines and threonines to alanines (*rif1-7A*) conferred increased temperature sensitivity to *hsk1-89* cells, as would be expected if this Rif1 protein had enhanced ability to interact with Sds21/Dis2.

We therefore proceeded to monitor the ability of these fission yeast Rif1 proteins to recruit Sds21 to telomeres and to the late origin *ars727*. Consistent with the idea that phosphorylation of the N terminus of Rif1 might downregulate its interaction with PP1, the *rif1-12D* allele displayed impaired recruitment of Sds21 to telomeres and to *ars727* (Figure 4F). The *rif1-7A* allele, on the other hand, led to a strong association of Sds21 at these sites, similar to wild-type. Further analysis will be needed to determine whether the profile of the association of Sds21 with chromatin during the cell cycle is affected in this mutant. Importantly, abrogating the RVxF and SILK domains in the context of the *rif1-7A* allele (*rif1-7APP1* allele) both restored the suppression of *hsk1-89* and impaired the interaction of Sds21 with chromatin, indicating that the synthetic lethality conferred by these alanine substitutions requires the ability of Rif1 to interact with PP1. Taken together, these results suggest that the interaction between Rif1 and PP1 is modulated by kinase activity on the Rif1 N terminus, likely by CDK and DDK.

### Conclusions

The key events in the activation of DNA replication are driven by phosphorylation (Labib, 2010). In particular, DDK-dependent phosphorylation of Mcm4 is a key regulatory event in the activation of the pre-RC complex (Tanaka et al., 2011). We show that this event is under control of Rif1-mediated phosphatase action, in agreement with two recent studies in budding yeast (Hiraga et al., 2014, Mattarocci et al., 2014). Our findings suggest that the action of kinases at origins is restricted not only upstream of their action (for example, at recruitment) but also after phosphorylation of their target(s) has occurred. This type of regulation might operate in addition, and in concert, with other modes of origin selection relying on nuclear domain architecture and chromatin accessibility. In this regard Rif1 might have a dual function in chromatin organization and as a recruiter of PP1 at these chromatin domains. Prevention of origin firing by Rif1 would ensure that the limiting factors required for origin activation would be reserved for preferential use at early-firing Rif1-free origins. The delay in firing at many early origins that is observed in the absence of Rif1/PP1 action in fission yeast could be a direct consequence of the scarce availability of limiting factors at early origins due to their increased utilization at misregulated late origins. Release of PP1-dependent inhibition of origin firing by the action of CDK and DDK on Rif1 could provide an additional layer of control on late origin firing and facilitate preferential activation later in the cell cycle at these origins.

Rif1 has a prominent role in orchestrating the replication program in both mouse and human cells (Yamazaki et al., 2012, Cornacchia et al., 2012). Given that mammalian Rif1 has also been shown to bind PP1 (Moorhead et al., 2008), it seems likely that the role of Rif1-dependent recruitment of PP1 in the control of DNA replication is a conserved feature of eukaryotes. Indeed, PP1 has recently been shown to reverse Cdc7 phosphorylation of MCM in *Xenopus* oocytes (Poh et al., 2014). It will be of interest to address whether PP1 binding might play a role in other processes regulated by Rif1 such as telomerase action and resection of double-strand breaks.

## Experimental Procedures

All strains and primers used are listed in Tables S1 and S2, respectively. Procedures for strain handling, construction, and synchronization and for protein extract preparation and analysis as well as for ChIP are given in the Supplemental Experimental Procedures.

## Supplemental Information

Document S1. Supplemental Experimental Procedures, Figures S1–S3, and Tables S1 and S2

Document S2. Article plus Supplemental Information
