# Supplementary material for: Protein Phosphatase 1 Recruitment by Rif1 Regulates DNA Replication Origin Firing by Counteracting DDK Activity
Source: Cell Rep. 2014 Mar 20;7(1):53–61. doi: 10.1016/j.celrep.2014.02.019 (PMC3989773; doi:10.1016/j.celrep.2014.02.019)
Supplement: Document S1. Supplemental Experimental Procedures, Figures S1–S3, and Tables S1 and S2 [file mmc1.pdf]

Cell Reports, Volume *7*

Supplemental Information

Protein Phosphatase 1 Recruitment by Rif1 Regulates DNA Replication Origin Firing by Counteracting DDK Activity

Anoushka Davé, Carol Cooley, Mansi Garg, and Alessandro Bianchi

Protein Phosphatase 1 recruitment by Rif1 regulates DNA replication origin firing by counteracting DDK activity

Anoushka Davé, Carol Cooley, Mansi Garg and Alessandro Bianchi

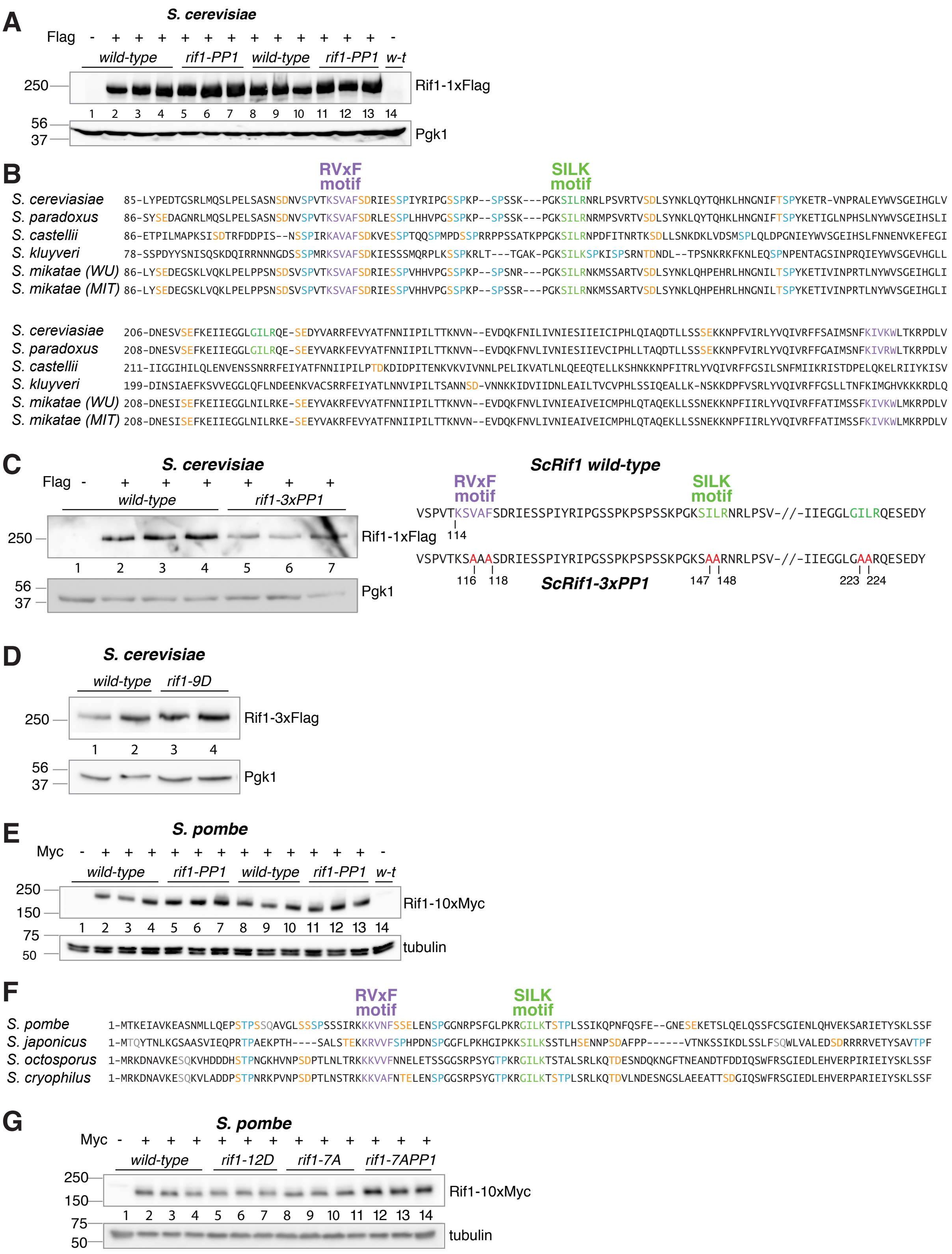

Figure S1. Related to Figures 1 and 4.
Expression levels of mutant Rif1 proteins.

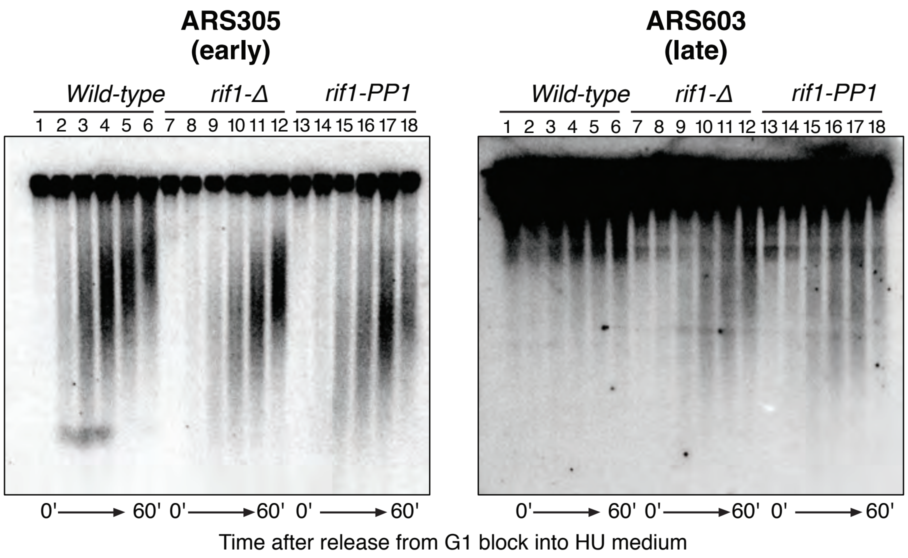

Figure S2. Related to Figure 2.
Loss of Rif1, or of Glc7 binding to Rif1, leads to early activation of late origin *ARS603*.

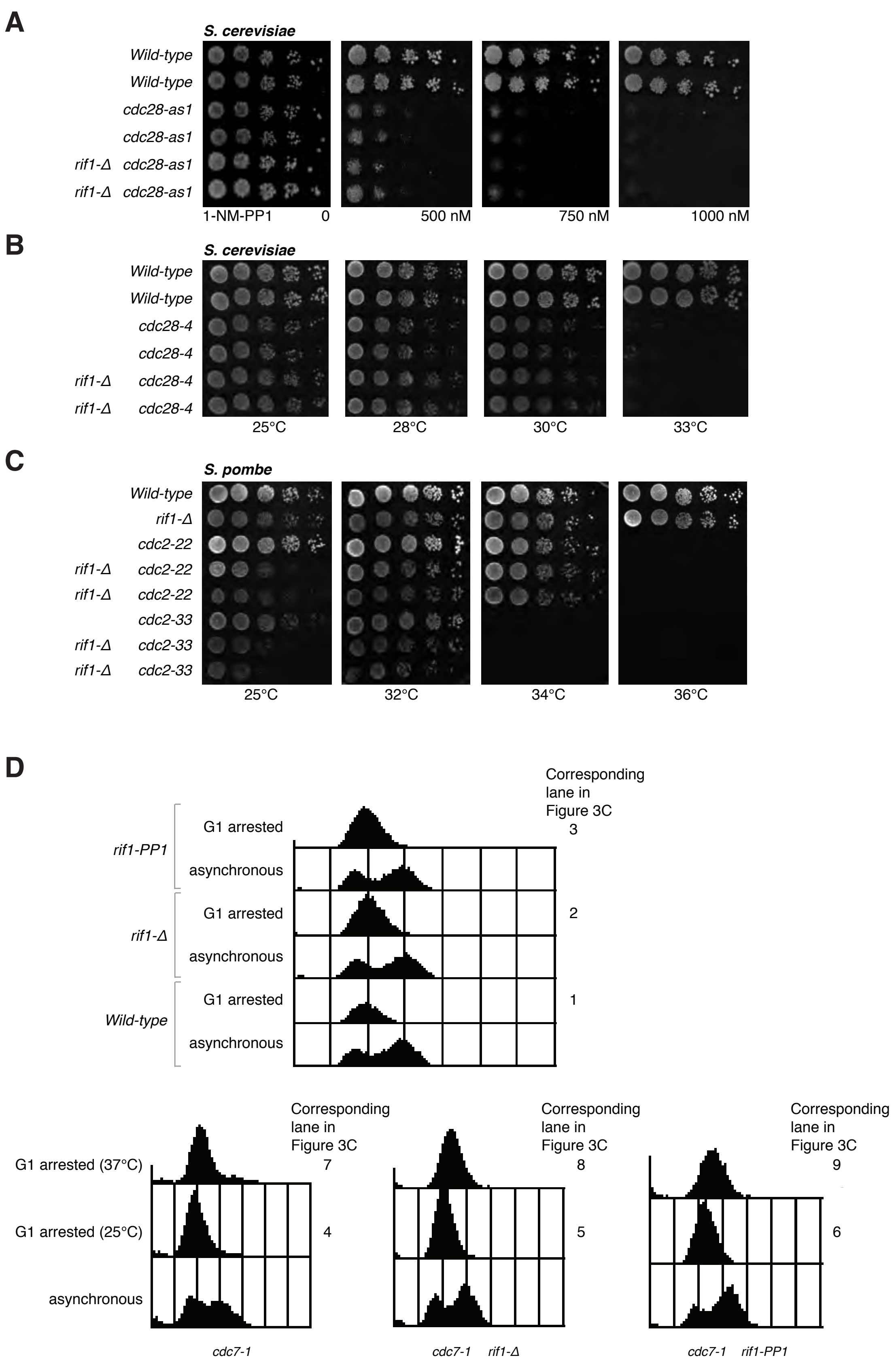

Figure S3. Related to Figure 3.

A-C. Loss of Rif1 does not suppress growth defect of several CDK alleles.

D. FACS analysis of samples from Figure 3C.

Supplemental Figure Legends

Figure S1. Expression levels of mutant Rif1 proteins. Related to Figure 1 and 4.

A. Analysis of protein levels of budding yeast wild-type Rif1 and Rif1-PP1 (referring to Figure 1) tagged at their C-terminus with 1 copy of the Flag epitope, from exponentially growing cultures. The same gel was also blotted for Pgk1, as a loading control. C-terminally tagged versions of the protein product of the *rif1-PP1* allele were found to be present at levels comparable to the wild-type tagged allele. **B.** Alignment of the relevant regions of Rif1 proteins from 5 *Saccharomyces* species (referring to Figure 4A). The RVxF and SILK type motifs are indicated in purple and green, respectively. Putative DDK sites are indicated in orange: these can be 'intrinsic' (SE/SD/TE/TD), or 'phosphorylation generated' where the negative charge C-terminal to the serine or threonine is provided by a prior phosphorylation event (for example by CDK). Finally, putative CDK sites are indicated in blue. **C.** Analysis of protein levels for the budding yeast Rif1 allele bearing changes to alanine at positions 116, 118, 147, 148, 223 and 224 (*rif1-3xPP1*). Although *S. cerevisiae* bears two additional SILK and RVxF type motifs further downstream from the first two (at positions 222 and 316), these putative motifs are embedded within the Ankyrin repeat region of the protein and are not well-conserved within the *Saccharomyces* genus. We found that the *rif1-3xPP1* allele (bearing mutations in the GILR motif at position 222 in addition to those within the first two motifs) was expressed at lower levels, possibly indicative of folding problems. **D.** Analysis of the protein levels of *S. cerevisiae* Rif1-9D, bearing the changes indicated in Figure 4A. **E.** Analysis of protein levels of fission yeast wild-type Rif1 and Rif1-PP1 (referring to Figure 1) tagged at their C-terminus with 10 copies of the Myc epitope, from exponentially growing cultures. The same gel was also blotted for tubulin, as a loading control. C-terminally tagged versions of the protein product of the *rif1-PP1* allele were found to be present at levels comparable to the wild-type tagged allele. **F.** Sequence alignment of the N-terminal region of the *rif1* gene in four *Schizosaccharomyces* species. RVxF (purple) and SILK (green) motifs, and putative DDK (orange) and CDK (blue) sites as above are indicated, in addition to Mec1/Tel1 sites

(grey). **G.** Analysis of the protein levels of *S. pombe* Rif1-12D, Rif1-7A, and Rif1-7APP1, bearing the changes indicated in Figure 4D.

Figure S2. Loss of Rif1, or of Glc7 binding to Rif1, leads to early activation of late origin ARS603. Related to Figure 2.

Analysis of DNA replication intermediates by alkaline agarose gel electrophoresis. Cells of the indicated genotypes from exponentially growing cultures in YPAD at 25°C were arrested in G1 phase of the cell cycle using 0.24 μM alpha-factor. The cells were then washed and released into S-phase in the presence of 200 mM HU, with time points harvested at 0, 20, 30, 40, 50 and 60 mins. Replication intermediates were then separated on alkaline denaturing agarose gels and analyzed by southern blotting using probes for ARS305 (left panel) and ARS603 (right panel). Probes were generated by PCR using oligos DO1787/1788 and DO2498/2499 respectively.

Figure S3. Loss of Rif1 does not suppress growth defect of several CDK alleles. Related to Figure 3.

A. Analysis of the budding yeast *cdc28-as1* (analog-sensitive) allele in the absence of *RIF1.* 5-fold serial dilutions of log-phase cultures of budding yeast strains of the indicated genotypes were plated onto YPAD media with increasing concentrations of the ATP analog 1-NM-PP1. Plates were imaged following 2-day incubations. **B.** Analysis of the suppression of the temperature-sensitivity phenotype of the budding yeast *cdc28-4* allele in the absence of *RIF1.* 5-fold serial dilutions of log-phase cultures of budding yeast strains of the indicated genotypes were plated onto YPAD and incubated at temperatures ranging from 25°C to 33°C. Plates were incubated for two days and then imaged. **C.** Analysis of temperature sensitive alleles of fission yeast *cdc2* combined with *RIF1* deletion. 10-fold serial dilutions of log-phase cultures of the indicated genotypes were spotted on rich medium and incubated for 3 days at temperatures ranging from 25°C to 36°C. **D.** FACS analysis of samples in Figure 3C. Samples were harvested from the same cell cultures analysed by western

blotting in Figure 3C and fixed in 70% ethanol before being stained with Propidium iodide. The distribution of DNA content was then measured using FACS and the profiles aligned using Cell Quest software (Becton Dickinson).

Table S1. List of strains.

Yeast	Strain	Figure	Genotype	Source
S. cerevisiae	YAB1661	1B, lanes 1, 2; 1D; 4C, lanes 1,2	MATα leu2 ura3 his3 GLC7-13myc::kanMX6	Kelly Tatchell
S. cerevisiae	YAB1680	1B, lanes 11, 12; 4C, lanes 11,12	matα-D ade2-D leu2::Gal-HO::LEU2 lys2-D Pol2-13Myc::HIS3 bar1::natMX6 adh4::pAB942 mnt2::LYS2 RIF1-FLAG::KANMX	This Study
S. cerevisiae	YAB1782	1B, lanes 3, 7; 4C, lane 3,7	MATα GLC7-13myc::kanMX6 RIF1-3xFLAG::URA3	This Study
S. cerevisiae	YAB1783	1B, lanes 4, 8; 4C, lane 4,8	MATα GLC7-13myc::kanMX6 RIF1-3xFLAG::URA3	This Study
S. cerevisiae	YAB1784	1B, lanes 5, 9	MATα GLC7-13myc::kanMX6 rif1-PP1-3xFLAG::URA3	This Study
S. cerevisiae	YAB1785	1B, lanes 6, 10	MATα GLC7-13myc::kanMX6 rif1-PP1-3FLAG::URA3	This Study
S. pombe	BAF364	1C lanes 1, 2; 1E; 4F	h- ura4-D18 leu1-32 sds21-EGFPN::ura4	Iain Hagan
S. pombe	BAF554	1C lanes 3, 5	h+ ura4-D18 leu1-32 sds21-EGFPN::ura4 his2- ade-	This Study
S. pombe	BAF558	1C lanes 4, 6	h+ sds21-EGFPN::ura4 rif1-PP1-10xMyc::leu1 ade6-216 ura4-D18 leu1-32	This Study
S. pombe	BAF366	1C lanes 7, 8; 1E; 1F	h- ura4-D18 leu1-32 dis2.NEGFP::ura4	Iain Hagan
S. pombe	BAF556	1C lanes 9, 11	h- ura4-D18 leu1-32 dis2.NEGFP::ura4 rif1-10xMyc::leu1	This Study
S. pombe	BAF561	1C lanes 10, 12	h+ dis2.NEGFP::ura4 rif1-PP1-10xMyc::leu1 ade6-216 his3-D1 ura4-D18 leu1-32	This Study
S. pombe	BAF552	1C lanes 13, 14; S1G, lane2	h+ rif1-10xMyc::leu1 ade6-216 his3-D1 ura4-D18 leu1-32	This Study
S. cerevisiae	YAB1709	1D	MATα GLC7-13myc::kanMX6 rif1-PP1	This Study
S. cerevisiae	YAB1710	1D	MATα GLC7-13myc::kanMX6 rif1-PP1	This Study
S. cerevisiae	YAB1711	1D	MATα GLC7-13myc::kanMX6 rif1-PP1	This Study
S. cerevisiae	YAB1712	1D	MATα GLC7-13myc::kanMX6 rif1::URA3	This Study
S. cerevisiae	YAB1714	1D	MATα GLC7-13myc::kanMX6 rif1::URA3	This Study
S. cerevisiae	YAB1715	1D	MATα GLC7-13myc::kanMX6 rif1::URA3	This Study
S. pombe	BAF365	1E	h+ ura4-D18 leu1-32 sds21-EGFPN::ura4 his2- ade-	Iain Hagan
S. pombe	BAF407	1E; 4F	h+ sds21-EGFPN::ura4 rif1-PP1 ade6-216 ura4-D18 leu1-32	This Study
S. pombe	BAF409	1E	h- sds21-EGFPN::ura4 Rif1::bsd ura4-D18 leu1-32	This Study
S. pombe	BAF410	1E	h- sds21-EGFPN::ura4 Rif1::bsd ade6-704 ura4-D18 leu1-32	This Study
S. pombe	BAF411	1E	h+ sds21-EGFPN::ura4 Rif1::bsd ade6-704 ura4-D18 leu1-32	This Study
S. pombe	BAF408	1F	h+ dis2.NEGFP::ura4 rif1-PP1 ade6-216 his3-D1 ura4-D18 leu1-32	This Study
S. pombe	BAF412	1F	h+ dis2.NEGFP::ura4 Rif1::bsd ade6-704 ura4-D18 leu1-32 nr1	This Study
S. pombe	BAF415	1F	h+ dis2.NEGFP::ura4 Rif1::bsd ade6-704 ura4-D18 leu1-32 nr2	This Study
S. pombe	BAF494	1F	h+ dis2.NEGFP::ura4 sds21::LEU2 ade6-216 his3-D1 ura4-D18 leu1-32	This Study
S. pombe	BAF495	1F	h- dis2.NEGFP::ura4 sds21::LEU2 ade6-216 ura4-D18 leu1-32	This Study
S. pombe	BAF496	1F	h+ dis2.NEGFP::ura4 sds21::LEU2 ura4-D18 leu1-32	This Study
S. pombe	BAF497	1F	h- dis2.NEGFP::ura4 sds21::LEU2 ade6-216 ura4-D18 leu1-32	This Study
S. cerevisiae	YAB1409	2A, left panels; 2B; S1A, lane 1, 14	matα-D ade2-D leu2::Gal-HO::LEU2 lys2-D Pol2-13Myc::HIS3 bar1::natMX6 adh4::pAB942 mnt2::LYS2	This Study
S. cerevisiae	YAB1410	2A, left panels	matα-D ade2-D leu2::Gal-HO::LEU2 lys2-D Pol2-13Myc::HIS3 bar1::natMX6 adh4::pAB704 mnt2::LYS2	This Study
S. cerevisiae	YAB1689	2A, center panels; 2B	matα-D ade2-D leu2::Gal-HO(LEU2) lys2-D Pol2-13Myc::HIS3 bar1::natMX6 adh4::pAB942 mnt2::LYS2 rif1-PP1	This Study
S. cerevisiae	YAB1706	2A, center panels	matα-D ade2-D leu2::Gal-HO(LEU2) lys2-D Pol2-13Myc::HIS3 bar1::natMX6 adh4::pAB704 mnt2::LYS2 rif1-PP1	This Study
S. cerevisiae	YAB1713	2A, center panels; 2B	matα-D ade2-D leu2::Gal-HO(LEU2) lys2-D Pol2-13Myc::HIS3 bar1::natMX6 adh4::pAB942 mnt2::LYS2 rif1-PP1	This Study
S. cerevisiae	YAB1618	2A, right panels; 2B	matα-D ade2-D leu2::Gal-HO(LEU2) lys2-D Pol2-13Myc::HIS3 bar1::natMX6 adh4::pAB942 mnt2::LYS2 rif1::TRP1	This Study
S. pombe	BAF392	2C	h- cdc25-22 cdc20-GFP:KanMX6 ura4-D18 leu1-32 ade6-216	This Study
S. pombe	BAF395	2C	h+ cdc25-22 cdc20-GFP:KanMX6 rif1-PP1 ura4-D18	This Study
S. pombe	BAF396	2C	h- cdc25-22 cdc20-GFP:KanMX6 rif1-PP1 ura4-D18	This Study
S. pombe	BAF401	2C	h+ rif1::bsd cdc25-22 cdc20-GFP:KanMX6 ade6-704 ura4-D18 leu1-32	This Study
S. pombe	BAF463	2C	h+ sds21::LEU2 cdc25-22 cdc20-GFP:KanMX6 ade6-216 his3-D1 ura4-D18 leu1-32	This Study
S. cerevisiae	YAB41	3A	MATα cdc7-1	Lab collection
S. cerevisiae	YAB1739	3A	MATα cdc7-1 rif1-PP1	This Study
S. cerevisiae	YAB1740	3A	MATα cdc7-1 rif1-PP1	This Study
S. cerevisiae	YAB1748	3A	MATα cdc7-1 rif1::TRP1	This Study
S. cerevisiae	YAB0	3A, S1C, lane 1; S2, lanes 1-6 both panels; S3B	MATa	Lab collection
S. cerevisiae	YAB1803	3A; S2, lane 7-13 both panels	MATa rif1::TRP1	This Study
S. cerevisiae	YAB1697	3A; S2, lanes 13–18 both panels	MATa rif1-PP1	This Study
S. pombe	BAF59	3B; S3C	h+ rif1::bsd ade6-704 ura4-D18 leu1-32	This Study
S. pombe	BAF394	3B	h+ rif1-PP1 ade6-216 his3-D1 ura4-D18 leu1-32	This Study
S. pombe	BAF400	3B; 4E	h- hsk1-89:ura4 ura4-D18 leu1-32	Tony Carr
S. pombe	BAF464	3B; 4E	h+ rif1::bsd hsk1-89:ura4 ura4-D18 leu1-32	This Study
S. pombe	BAF484	3B; 4E	h- rif1-PP1 hsk1-89:ura4 ura4-D18 leu1-32	This Study
S. pombe	BAF6	3B; S1E; S1G; S3C	h+ ade6-216 his3-D1 ura4-D18 leu1-32	This Study
S. cerevisiae	YAB1799	3C, lane 1; S3D	MATa mcm4-1xFlag::kanMX	This Study
S. cerevisiae	YAB1805	3C, lane 2; S3D	MATa Rif1::TRP1 Mcm4-1xFlag::kanMX	This Study
S. cerevisiae	YAB1801	3C, lane 3; S3D	MATa rif1-PP1 mcm4-1xFlag::kanMX	This Study
S. cerevisiae	YAB1913	3C, lane 4, 7; S3D	MATa cdc7-1 mcm4-1xFlag::kanMX	This Study

S. cerevisiae	YAB1888	3C, lane 5, 8; S3D	MATa cdc7-1 Rif1::TRP1 Mcm4-1xFlag::kanMX	This Study
S. cerevisiae	YAB1904	3C, lane 6, 9; S3D	MATa cdc7-1 rif1-PP1 mcm4-1xFlag::kanMX	This Study
S. cerevisiae	YAB1902	4B	MATa rif1-9D	This Study
S. cerevisiae	YAB1832	4B	MATα cdc7-1 rif1-9D	This Study
S. cerevisiae	YAB1834	4B	MATα cdc7-1 rif1-9D	This Study
S. cerevisiae	YAB1875	4C	MATα GLC7-13myc::kanMX6 rif1-9D-3xFLAG::URA3	This Study
S. cerevisiae	YAB1877	4C	MATα GLC7-13myc::kanMX6 rif1-9D-3xFLAG::URA3	This Study
S. pombe	BAF548	4E	h+ rif1-12D ade6-216 his3-D1 ura4-D18 leu1-32	This Study
S. pombe	BAF550	4E	h+ rif1-7A ade6-216 his3-D1 ura4-D18 leu1-32	This Study
S. pombe	BAF551	4E	h+ rif1-7APP1 ade6-216 his3-D1 ura4-D18 leu1-32	This Study
S. pombe	BAF604	4E	h- hsk1-89:ura4 rif1-12D ade6-216 ura4-D18 leu1-32	This Study
S. pombe	BAF605	4E	h+ hsk1-89:ura4 rif1-12D his3-D1 ura4-D18 leu1-32	This Study
S. pombe	BAF606	4E	h- hsk1-89:ura4 rif1-7A his3-D1 ura4-D18 leu1-32	This Study
S. pombe	BAF607	4E	h+ hsk1-89:ura4 rif1-7A ade6-216 his3-D1 ura4-D18 leu1-32	This Study
S. pombe	BAF608	4E	h- hsk1-89:ura4 rif1-7APP1 ura4-D18 leu1-32	This Study
S. pombe	BAF609	4E	h+ hsk1-89:ura4 rif1-7APP1 ade6-216 his3-D1 ura4-D18 leu1-32	This Study
S. pombe	BAF621	4F	h+ sds21-EGFPN::ura4 rif1-12D ade6-216 his3-D1 ura4-D18 leu1-32	This Study
S. pombe	BAF622	4F	h- sds21-EGFPN::ura4 rif1-12D ura4-D18 leu1-32	This Study
S. pombe	BAF624	4F	h- sds21-EGFPN::ura4 rif1-7A his3-D1 ura4-D18 leu1-32	This Study
S. pombe	BAF625	4F	h+ sds21-EGFPN::ura4 rif1-7APP1 his3-D1 ura4-D18 leu1-32	This Study
S. pombe	BAF626	4F	h- sds21-EGFPN::ura4 rif1-7APP1 ade6-216 ura4-D18 leu1-32	This Study
S. cerevisiae	YAB1703	S1A, lane 11	matα-D ade2-D leu2::Gal-HO::LEU2 lys2-D Pol2-13Myc::HIS3 bar1::natMX6 adh4::pAB942 mnt2::LYS2 RIF1-1xFLAG::kanMX rif1-PP1	This Study
S. cerevisiae	YAB1704	S1A, lane 12	matα-D ade2-D leu2::Gal-HO::LEU2 lys2-D Pol2-13Myc::HIS3 bar1::natMX6 adh4::pAB942 mnt2::LYS2 RIF1-1xFLAG::kanMX rif1-PP1	This Study
S. cerevisiae	YAB1705	S1A, lane 13	matα-D ade2-D leu2::Gal-HO::LEU2 lys2-D Pol2-13Myc::HIS3 bar1::natMX6 adh4::pAB942 mnt2::LYS2 RIF1-1xFLAG::kanMX rif1-PP1	This Study
S. cerevisiae	YAB1678	S1A, lane 2-4; S1C, lane 2-4	matα-D ade2-D leu2::Gal-HO::LEU2 lys2-D Pol2-13Myc::HIS3 bar1::natMX6 adh4::pAB942 mnt2::LYS2 RIF1-1xFLAG::kanMX	This Study
S. cerevisiae	YAB1700	S1A, lane 5	matα-D ade2-D leu2::Gal-HO::LEU2 lys2-D Pol2-13Myc::HIS3 bar1::natMX6 adh4::pAB942 mnt2::LYS2 RIF1-1xFLAG::kanMX rif1-PP1	This Study
S. cerevisiae	YAB1701	S1A, lane 6	matα-D ade2-D leu2::Gal-HO::LEU2 lys2-D Pol2-13Myc::HIS3 bar1::natMX6 adh4::pAB942 mnt2::LYS2 RIF1-1xFLAG::kanMX rif1-PP1	This Study
S. cerevisiae	YAB1702	S1A, lane 7	matα-D ade2-D leu2::Gal-HO::LEU2 lys2-D Pol2-13Myc::HIS3 bar1::natMX6 adh4::pAB942 mnt2::LYS2 RIF1-1xFLAG::kanMX rif1-PP1	This Study
S. cerevisiae	YAB1679	S1A, lane 8-10	matα-D ade2-D leu2::Gal-HO::LEU2 lys2-D Pol2-13Myc::HIS3 bar1::natMX6 adh4::pAB942 mnt2::LYS2 RIF1-1xFLAG::kanMX	This Study
S. cerevisiae	YAB1681	S1C, lane 5	matα-D ade2-D leu2::Gal-HO::LEU2 lys2-D Pol2-13Myc::HIS3 bar1::natMX6 adh4::pAB942 mnt2::LYS2 RIF1-1xFLAG::kanMX rif1-3xPP1	This Study
S. cerevisiae	YAB1682	S1C, lane 6	matα-D ade2-D leu2::Gal-HO::LEU2 lys2-D Pol2-13Myc::HIS3 bar1::natMX6 adh4::pAB942 mnt2::LYS2 RIF1-1xFLAG::kanMX rif1-3xPP1	This Study
S. cerevisiae	YAB1683	S1C, lane 7	matα-D ade2-D leu2::Gal-HO::LEU2 lys2-D Pol2-13Myc::HIS3 bar1::natMX6 adh4::pAB942 mnt2::LYS2 RIF1-1xFLAG::kanMX rif1-3xPP1	This Study
S. cerevisiae	YAB1843	S1D, lane 1	MATa Rif1-3Flag	This Study
S. cerevisiae	YAB1844	S1D, lane 2	MATa Rif1-3Flag	This Study
S. cerevisiae	YAB1841	S1D, lane 3	MATα Rif1-3Flag, rif1-9D	This Study
S. cerevisiae	YAB1842	S1D, lane 4	MATα Rif1-3Flag, rif1-9D	This Study
S. pombe	BAF486	S1E	h+ rif1-10xMyc::ura4 ade6-216 his3-D1 ura4-D18 leu1-32	This Study
S. pombe	BAF487	S1E	h+ rif1-PP1-10xMyc::ura4 ade6-216 his3-D1 ura4-D18 leu1-32	This Study
S. pombe	BAF488	S1E	h+ rif1-PP1-10xMyc::ura4 ade6-216 his3-D1 ura4-D18 leu1-32	This Study
S. pombe	BAF489	S1E	h+ rif1-PP1-10xMyc::ura4 ade6-216 his3-D1 ura4-D18 leu1-32	This Study
S. pombe	BAF490	S1E	h+ rif1-PP1-10xMyc::ura4 ade6-216 his3-D1 ura4-D18 leu1-32	This Study
S. pombe	BAF491	S1E	h+ rif1-PP1-10xMyc::ura4 ade6-216 his3-D1 ura4-D18 leu1-32	This Study
S. pombe	BAF492	S1E	h+ rif1-PP1-10xMyc::ura4 ade6-216 his3-D1 ura4-D18 leu1-32	This Study
S. pombe	BAF553	S1G, lane 3, 4	h+ rif1-10xMyc::leu1 ade6-216 his3-D1 ura4-D18 leu1-32	This Study
S. pombe	BAF618	S1G, lane 5, 6, 7	h+ rif1-12D-10xMyc::leu1 ade6-216 his3-D1 ura4-D18 leu1-32	This Study
S. pombe	BAF619	S1G, lane 8 ,9, 10	h+ rif1-7A-10xMyc::leu1 ade6-216 his3-D1 ura4-D18 leu1-32	This Study
S. pombe	BAF620	S1G, lane 11, 12, 13	h+ rif1-7APP1-10xMyc::leu1 ade6-216 his3-D1 ura4-D18 leu1-32	This Study
S. cerevisiae	YAB1910	S3A	SK1 MATa ho:LYS2 lys2 ura3 leu2::hisG his3::hisG trp1::hisG	Hideo Tsubouchi
S. cerevisiae	YAB1847	S3A	SK1 MATα ho::LYS2 lys2 ura3 arg4-nsp leu2::hisG cdc28as1	Matt Neale
S. cerevisiae	YAB1900	S3A	SK1 MATα ho::LYS2 lys2 ura3 arg4-nsp leu2::hisG cdc28as1 rif1::TRP1	This Study
S. cerevisiae	YAB1868	S3B	MATa cdc28-4	Jorrit Enserink
S. cerevisiae	YAB1914	S3B	MATa cdc28-4 rif1::TRP1	This Study
S. pombe	BAF591	S3C	h- cdc2-22	Tony Carr
S. pombe	BAF612	S3C	h+ cdc2-22 rif1::bsd ura4-D18 leu1-32	This Study
S. pombe	BAF613	S3C	h- cdc2-22 rif1::bsd ade6-704 ura4-D18	This Study
S. pombe	BAF590	S3C	h- cdc2-33 ade6-704 leu1-32 ura4-D18	Tony Carr
S. pombe	BAF614	S3C	h- cdc2-33 ade6-704 ura4-D18 leu1-32	This Study
S. pombe	BAF615	S3C	h+ cdc2-33 ade6-704 ura4-D18 leu1-32	This Study

NOTES: In the strains bearing the Gal-HO cassette there is no HO site in the genome (the endogenous site at the MAT locus has been deleted) and therefore no HO cleavage is induced, and no effect on cell cycle progress is elicited. pAB942 introduces an ADE2 marker and a stretch of telomeric repeats at the subtelomere of VII-L, with no effects on the other loci and telomeres analyzed. pAB704 (present in strains YAB1410 and YAB1706) is similar to pAB942 but carries an HO site: this plasmid will introduce a DSB at the ADH4 locus, with no consequences on cell cycle progression or replication timing of other loci, as the break is flanked by telomeric arrays.

Table S2. List of oligonucleotides.

Oligo name	Purpose	Name of target locus/template	Oligo sequence
DO1344	QPCR	Sp TEL (subtelomeric)	TATTTCTTTATTCAACTTACCGCACTTC
DO1348	QPCR	Sp TEL (subtelomeric)	CAGTAGTGCAGTGTATTATGATAATTAAAATGG
DO2374	QPCR	Sp non-ori1	TTCAGCGCAAGAACTATTGG
DO2375	QPCR	Sp non-ori1	CACATAACCCGCTAGCTTCC
DO2391	QPCR	Sp 3ori333	AAAATGCCTTGTGCTTTGTG
DO2392	QPCR	Sp 3ori333	GGCCGATCGTCCAGAAATTA
DO2393	QPCR	Sp 3ori1283	GCAGAAGCAGAAGTTCAAAGG
DO2394	QPCR	Sp 3ori1283	AACCAGATTCTTCGGTGCAG
DO2395	QPCR	Sp ars727	CCCCACAAGGTACGAAAAAG
DO2396	QPCR	Sp ars727	TACTCATTTCCCCCACCTCA
DO2401	QPCR	Sp AT2035	TGGTACGTCGAGTGAGACACA
DO2402	QPCR	Sp AT2035	CACTCCGGGGAAGAGGTTAT
DO2406	QPCR	Sp cen1	TCAATTCTGTAAGTTTGCTGTGC
DO2407	QPCR	Sp cen1	AGGAAACGCCATGGAGTACA
DO2449	QPCR	Sp ars2004	GATTGACTCAGTACACACCACACA
DO2451	QPCR	Sp ars2004	GCAATTGTGATGGAATTGGTT
DO2459	QPCR	Sp 2ori326	GGAAATCGAGCAGAGGTCAG
DO2460	QPCR	Sp 2ori326	TTGACGTTGTGCTAAAAGGTG
DO2463	QPCR	Sp 2ori4451	ACGATGTCATTTGGCACTCA
DO2464	QPCR	Sp 2ori4451	AAAAATTTGGAACACTGCTTGT
DO1230	Deletion of Sp rif1	pAB742	AAACTTATTTGTGCAATTTTTTGGTGCGATCGGTCCCATGAAGCTAAAACATTTTTAAAAGCATAACAGCTGATGTTGCGCACTCAACCCTATCTCGG
DO1231	Deletion of Sp rif1	pAB742	GAACCCCATTAATAAGATTGATTTATGACTAAATTGACCCCAATGCCGGTCCGTAATTCCTGTACCCATGAGTTGAATAACTCGAAATTAACCCTCAC
DO2444	genotyping of Sp rif1-PP1	Sp rif1	AACCTTCAACCCCATCATCA
DO2446	genotyping of Sp rif1-PP1/-12D/-7A/-7APP1	Sp rif1	CCTCGGGAAGACTTGGTGTA
DO2502	genotyping of Sp rif1-12D/-7A/-7APP1	Sp rif1	GATCGGTCCCATGAAGCTAA
DO1135	QPCR	Sc TEL VI-R	CCATGACCCAGTCCTCATTT
DO1136	QPCR	Sc TEL VI-R	TGGCAAGGGTAAAAACCAGT
DO1138	QPCR	Sc ARS607	CTTTAGCTGGGTTTATGGGAGGTT
DO2517	QPCR	Sc ARS607	TAATGCACGAGCCGAAACAA
DO1232	QPCR	Sc TEL XV-L	CCTTACCTCCCCACTCGTTAC
DO2518	QPCR	Sc TEL XV-L	ATCGTGGTTCGCTGTGGTAT
DO2519	QPCR	Sc ARS522	AAGCAAATTGCAGAAGGTTATGAA
DO2520	QPCR	Sc ARS522	TTCAAGGCTCTAGCATATGAAACG
DO2208	QPCR	Sc ARS1412	AAGCAAATTGCAGAAGGTTATGAA
DO2209	QPCR	Sc ARS1412	TTCAAGGCTCTAGCATATGAAACG
DO998	QPCR	Sc ARS603	AATCCACCACAAAGCCCTAA
DO999	QPCR	Sc ARS603	CGAGGGTCGAAATCATCATC
DO373	RIF1 C-terminal FLAG tagging	pAB1090	ATTTTATGATGAGGCTCGAATATTACTCAAACAGGGATAATGATATGAATCGGATCCCCGGGTTAATTAA
DO374	RIF1 C-terminal FLAG tagging	pAB1090	ATTGTAATTAATTTATTGCCATTTTGATCTATTCTACATACTAACAATCAGAATTCGAGCTCGTTTAAAC
DO1734	MCM4 C-terminal FLAG tagging	pAB1090	TTGTCCTTGGCGAGGGTGTAAGGAGATCAGTTCGCCTGAATAACCGTGTCGGGGGAGGCGGGGGTGGA
DO1735	MCM4 C-terminal FLAG tagging	pAB1090	TTAGTATTTATTAATTGTTACGCAGGGAATGATTGTAGTAGACAGCATCAGAATTCGAGCTCGTTTAAAC
DO2667	Genotyping of Sc rif1-PP1	Sc RIF1	AGGAAGCAGGCTAATGCAAA
DO2668	Genotyping of Sc rif1-PP1	Sc RIF1	ACTCTAAGGCGCGAGGATTT
DO1787	PCR of ARS305 probe	ARS305	ATCGTGTAAGCTGGGGTGAC
DO1788	PCR of ARS305 probe	ARS305	GGCAAACGTCCAAAGACAAT
DO2498	PCR of ARS603 probe	ARS603	GGTATTGCTGTTTTAAGTGAG
DO2499	PCR of ARS603 probe	ARS603	CATAGATATCGGGTTACTAAAG

Supplemental Experimental Procedures

Strains and plasmids

All budding yeast strains were generated in the W303 background (*MATa ade2-1 his3-11,15 leu2-3,112 trp1-1 ura3-1 can1-100 RAD5*). A complete list of the strains used is reported in Table S1. Standard budding yeast handling and growth conditions were used. Rich medium was YPAD, and drop-out media were made using pre-made mixes from United States Biological.

Fission yeast strains (containing *ade6-M216 ura4-D18 leu1-32 his3-D1* or combinations thereof) were grown in YES rich medium (Moreno et al., 1991) or YNG minimal medium (2% w/v glucose, 30 mM glutamate, 0.17% w/v YNB mix without amino acids ammonium sulphate or thiamine (United States Biological), 0.53% w/v SC dropout mix (United States Biological), 2% w/v agar, pH 6.0. Transformations were performed using a modified version of the protocol described by Bahler (Bahler et al., 1998): cells were incubated with plasmid DNA, carrier DNA and 40% PEG/LiAc/TE solution for 2 hrs at 30°C, before a 10 min heat shock at 42°C; transformations were subsequently plated out directly onto plates without centrifugation.

C-terminal 1xFlag-tagging of ScRif1 and ScMcm4 was obtained with PCR products generated from pFA6a-6xGLY-1xFLAG-kanMX6 (pAB1090) using oligos DO373/DO374 and DO1734/DO1735 respectively. Alternatively, ScRif1 was C-terminally 3xFlag-tagged using a MfeI-linearized plasmid containing the protein's C-terminus cloned in-frame to a triple Flag tag linked by an 8xGlycine linker (pAB1750). Sc *RIF1* gene deletions were obtained using plasmids pAB1511 (TRP1), pAB1655 (URA3), or pAB1749 (ADE2) linearized with SphI. Integration of the Sc *rif1-PP1* allele was obtained by transformation with pAB1654 linearized with SgrAI: colonies were then streaked on 5-FOA and genotyped by PCR with DO2267/DO2268 (314 bp product) followed by restriction enzyme digest with PstI or PvuII (PstI digestion: 314 bp on *RIF1*, 227 + 87 bp on *rif1-PP1*; PvuII digestion: 314 bp on *RIF1*, 146 + 90 + 78 bp on *rif1-PP1*). The *rif1-9D* allele was made in the same way as for *rif1-PP1* allele: SgrAI digested pAB1752 was used for the initial integration followed by FOA pop-out.

Genotyping was done by PCR using the same primers as for *rif1-PP1* (DO2267/DO2268) and digested with AgeI (*RIF1*: 253 + 61 bp, *rif1-9D*: uncut).

To obtain the Sp *rif1-PP1* allele, the same procedure was used using plasmid pAB1664 linearized with BglII. SacI digestion of a 360 bp PCR product made with DO2444/DO2446 on genomic DNA indicated the presence of the allele (237 + 123 bp in *rif1-PP1*; uncut in WT). To obtain the *rif1-12D/-7A/-7APP1* alleles, BglII-linearized plasmids (pAB1741, 1740, 1742 respectively) containing the mutated region and a *ura*+ marker were transformed into yeast. The resultant colonies were counter-selected on 5'-fluoroorotic acid (5'-FOA) to select for recombination at the Sp *rif1* locus. Fission yeast strains with mutations in putative CDK/DDK phosphorylation sites were verified by PCR using DO2502/DO2446 which results in a 462 bp product. DraIII cleaves *rif1-7A* and *rif1-7APP1* but not *rif1-12D* PCR products to give 260 + 202 bp fragments, while ApoI cleaves *rif1-7A* and *rif1-12D* to give 176, 175 and 111bp fragments, and *rif1-7APP1* to give 287 + 175 bp fragments. All Rif1 mutant alleles were generated by gene synthesis (Eurofins). The C-terminus of Sp Rif1 was tagged with 10xMyc epitopes using pAB1462 or pAB1744 linearized with MfeI. Sp *rif1*+ was deleted using a PCR product made with oligonucleotides DO1230/DO1231 on using pSVEM-bsd (pAB742) as template (Erler et al., 2006). Fission yeast strains with deletions of *sds21*+ or *dis2+,* or with N-terminal EGFP-tags of the same genes expressed from their chromosomal locus were kindly gifted by Iain Hagan. The *hsk1-89* strain was gratefully received from Tony Carr.

Immunoprecipitation of ScRif1

Cultures of exponentially growing budding yeast cells (100 ml of 1 x 10^7 cells/ml) were lysed in 15 mM Hepes pH 7.6, 150 mM NaCl, 0.5 % NP-40 with three 20 s pulses in a Beadbeater. The lysate was clarified by centrifugation and the supernatant was incubated first with anti-myc 9E10 monoclonal antibody for 2 hours at 4°C and then with Protein G Dynabeads for 1 hour at 4°C. Bound proteins (eluted by boiling in Laemmli buffer) and input samples were separated on a 8% (top panel) and 10% (bottom panel) SDS gel and Western blotting was performed using anti-Flag (M2, Sigma) or anti-Myc antibody (homemade 9E10).

Immunoprecipitation of SpRif1

Whole cell extracts from $2x10^8$ fission yeast were prepared by resuspending in 500 µl of chilled lysis buffer (50 mM NaCl, 50 mM Tris pH 7.5, 10% glycerol, 4 mM B-mercaptoethanol, 1 mM EDTA) containing 1 complete protease inhibitor tablet (Roche) per 7 ml of buffer. Following lysis in a beadbeater for 3 min, 1 µl Benzonase Nuclease (Novazyme) and 50 µl 10% NP40 were added to the lysate and incubated for 30 min on ice. After centrifugation for 10 min, 30 µl of the cleared lysate was boiled with 10 µl 4x Laemmli buffer and kept aside as Input. Protein G Dynabeads (Invitrogen) were blocked for 30 min in lysis buffer + 5% BSA. 25 µl of the beads were used to pre-clear the remaining lysate for 30 min at 4°C on a rotating wheel. 1 µl rabbit anti-GFP antibody (Invitrogen) was added to the supernatant obtained after magnetic separation of the beads. The samples were then incubated with rotation at 4°C for 1 hr. 25 µl of Dynabeads were added before further incubation at 4°C for 2 hrs. Following magnetic separation and removal of the supernatant, the beads were resuspended in 40 µl 1x Laemmli buffer and boiled for 5 min.

Synchronization of budding yeast cultures

Budding yeast cells were grown in 100 ml overnight cultures in the appropriate drop-out SC medium containing 4% raffinose. The cultures were then diluted into 300 ml of YPA 4% raffinose and grown for 2 hours with 0.025 µM α-factor to arrest the cells in G1 phase of the cell cycle. Cells at a density of 1×10^7 cells/ml were then switched to YPA 4% galactose for 4 hours at 30°C, while maintaining the arrest with 0.025 µM α-factor. Cells were released into S-phase by washing twice with water and switching the cells to YPA+GAL containing 0.125 mg/ml pronase at 18°C.

Quantification of budding and fission yeast DNA

Fission yeast strains containing the *cdc25-22* allele were grown to mid-log-phase at 25°C, arrested in G2 for 3h at 36°C and subsequently released into medium containing 25 mM HU at 25°C for 140 min. Samples were collected for G2-arrested cells and S-phase-arrested cells (140 min in HU) by cross-linking cells in 1% formaldehyde.

Budding yeast strains were synchronized as described above and samples collected after crosslinking in 1% formaldehyde.

For both yeasts, cell were lysed on a BeadBeater and centrifuged. Pellets were then resuspended in 500 µl ChIP Lysis buffer (50 mM HEPES pH 7.5,140 mM NaCl, 1 mM EDTA, 1% Igepal CA-630, 0.1% sodium deoxycholate) and sonicated 15 x 30 sec on high in a Diagenode Bioruptor. 10 µl of the supernatant collected after centrifugation was added to 110 µl TES (20 mM Tris-Cl pH7.5, 1 mM EDTA, 1% SDS) and de-crosslinked overnight at 65°C. DNAs were purified using the Qiagen PCR purification kit.

ChIP

ChIP was performed as described previously (Bianchi and Shore, 2007). Briefly, after cross-linking in 1% formaldehyde, cells were lysed and sonicated to achieve DNA fragments <500 bp. Immunoprecipitations were carried out with anti-Myc 9E10 (supernatant from a 9E10 hybridoma cell-line) or anti-GFP (Invitrogen) and ProteinG Dynabeads (Invitrogen) against N- or C-terminally tagged proteins, as indicated. Both an aliquot of sonicated cleared extract (input) and the immunoprecipitated material were de-cross-linked in TE plus 1% SDS at 65°C overnight. Quantitation of immunoprecipitated DNA was obtained by real-time PCR using SYBR Green detection on a Roche Light Cycler 480 II instrument and expressed either as percent of starting (input) material or as fold-enrichment over a control locus (Pfaffl, 2001). Primers used are listed in Table S2.

Protein analysis by Western blotting

Protein extracts were made using asynchronous cultures by lysing cells using 0.5 mm glass beads and 200 µl 20% TCA in a Bead-Beater. Next, lysates were added to 400 µl 5% TCA. Cells were then pelleted and resuspended in 200 µl Laemmli sample buffer (250 mM TrisCl pH7.5, 2% SDS, 5% Glycerol, 0.1% BromoPhenol Blue, 2.5% β-mercaptoethanol) and boiled at 95°C for 5min. The samples were then separated on SDS-PAGE gel of required percentage and western blotting was

performed. The proteins were visualized by treating the membrane with ECL and imaged using a LAS 4000 instrument (GE).

Analysis of Mcm4 phosphorylation

Budding yeast cells were grown in 5 ml overnight cultures in YPAD at 25°C (wild-type*, rif1-Δ, rif1-PP1, cdc7-1, cdc7-1 rif1-Δ, cdc7-1 rif1-PP1*). The cultures were then diluted to 0.4×10^7 cells/ml in 10 ml YPAD and were grown at 25°C for one cell cycle to obtain a log phase culture. 2.4 μM α-factor was added to arrest the cells in G1 phase of the cell cycle at either 25°C or 37°C for 1 hour. After 1 hour the cells were pelleted and resuspended in fresh 10 ml YPAD with 2.4 μM α-factor and incubated for another hour at the respective temperatures. After a total of 2 hours the cells were harvested and were resuspended in 600 μl 100%TCA and were kept on ice for 10 minutes. The cells were pelleted by centrifuging at 3000 rpm for 2 minutes, followed by two acetone washes. The pellets were then dried under vacuum and were resuspended in 100 μl Urea buffer (50 mM Tris-Cl pH 7.5, 5 mM EDTA, 6 M Urea, 1% SDS). 200 μl 0.5 mm glass beads were added to the tubes and the cells were lysed in a bead beater 5 times for 45 seconds with 1 min on ice in between. The extracts were incubated at 65°C for 10 minutes and centrifuged at 14000 rpm for 10 minutes before the addition of 200 μl of 2x Laemmli buffer. Samples were boiled for 5 min and separated on 6% SDS-PAGE gel.

Alkaline gels

DNA replication intermediates were analyzed using alkaline agarose gel electrophoresis. Briefly, DNA was prepared by lysing 1×10^8 cells using zymolyase 100T (USB) extraction method and the DNA was then separated on a 1% denaturing alkaline gel (50 mN NaOH, 1 mM EDTA). Southern blotting was then performed and the DNA was probed with ^{32}P-labelled ARS305 probe and ARS603 probe. Probes were generated by PCR using oligos DO1787/1788 and DODO2498/DO2499.

FACS analysis

Cells were fixed in 70% ethanol and treated with 200 μg/ml RNAse. Cells were then stained

with 10 µg/ml Propidium Iodide (PI) before being analyzed in 50 mM Na citrate 10 µg/ml PI on a Becton Dickinson FACScalibur flow cytometer with CellQuest software.

Supplemental References

Bahler, J., Wu, J.Q., Longtine, M.S., Shah, N.G., McKenzie, A.,.3., Steever, A.B., Wach, A., Philippsen, P., and Pringle, J.R. (1998). Heterologous modules for efficient and versatile PCR-based gene targeting in Schizosaccharomyces pombe. Yeast 14, 943-951.

Bianchi, A., and Shore, D. (2007). Early replication of short telomeres in budding yeast. Cell 128, 1051-1062.

Erler, A., Maresca, M., Fu, J., and Stewart, A.F. (2006). Recombineering reagents for improved inducible expression and selection marker re-use in Schizosaccharomyces pombe. Yeast 23, 813-823.

Moreno, S., Klar, A., and Nurse, P. (1991). Molecular genetic analysis of fission yeast Schizosaccharomyces pombe. Methods Enzymol 194, 795-823.

Pfaffl, M.W. (2001). A new mathematical model for relative quantification in real-time RT-PCR. Nucleic Acids Res 29, e45.
