# Supplementary material for: Protein Phosphatase 1 Recruitment by Rif1 Regulates DNA Replication Origin Firing by Counteracting DDK Activity
Source: Cell Rep. 2014 Mar 20;7(1):53–61. doi: 10.1016/j.celrep.2014.02.019 (PMC3989773; doi:10.1016/j.celrep.2014.02.019)
Supplement: Document S2. Article plus Supplemental Information [file mmc2.pdf]

Cell Reports
Report

OPEN ACCESS
CellPress

Protein Phosphatase 1 Recruitment by Rif1 Regulates DNA Replication Origin Firing by Counteracting DDK Activity

Anoushka Davé,[1,2] Carol Cooley,[1,2] Mansi Garg,[1] and Alessandro Bianchi[1,*]
[1]Genome Damage and Stability Centre, School of Life Sciences, University of Sussex, Brighton BN1 9RQ, UK
[2]These authors contributed equally to this work
*Correspondence: a.bianchi@sussex.ac.uk
http://dx.doi.org/10.1016/j.celrep.2014.02.019
This is an open access article under the CC BY license (http://creativecommons.org/licenses/by/3.0/).

SUMMARY

The firing of eukaryotic origins of DNA replication requires CDK and DDK kinase activities. DDK, in particular, is involved in setting the temporal program of origin activation, a conserved feature of eukaryotes. Rif1, originally identified as a telomeric protein, was recently implicated in specifying replication timing in yeast and mammals. We show that this function of Rif1 depends on its interaction with PP1 phosphatases. Mutations of two PP1 docking motifs in Rif1 lead to early replication of telomeres in budding yeast and misregulation of origin firing in fission yeast. Several lines of evidence indicate that Rif1/PP1 counteract DDK activity on the replicative MCM helicase. Our data suggest that the PP1/Rif1 interaction is downregulated by the phosphorylation of Rif1, most likely by CDK/DDK. These findings elucidate the mechanism of action of Rif1 in the control of DNA replication and demonstrate a role of PP1 phosphatases in the regulation of origin firing.

INTRODUCTION

The replication of Eukaryotic genomes is a highly regulated process. DNA replication starts at defined positions in the genome, called origins, the activation of which is strictly confined to the S phase of the cell cycle (Labib, 2010). Binding of the heterohexameric MCM helicase to a DNA-bound origin recognition complex (ORC) constitutes a first step in the assembly of a functional origin complex, or prereplication complex (pre-RC). The pre-RC is then activated by the action of the cyclin- and Dbf4-dependent kinases (CDK and DDK, respectively) at the end of the G1 phase. The essential function of CDK in DNA replication is the phosphorylation of the Sld2 and Sld3 proteins (Tanaka et al., 2007; Zegerman and Diffley, 2007), whereas the main role of DDK appears to be the phosphorylation of the MCM helicase, particularly the Mcm4 subunit (Sheu and Stillman, 2010). MCM phosphorylation allows recruitment of Cdc45/Sld3 and the GINS complex, which immediately precede polymerase loading and replication start (Heller et al., 2011; Tanaka et al., 2011).

However, these events do not take place simultaneously at all origins at the outset of S phase but are strictly choreographed, with origins being activated in a defined sequence that is a characteristic of each genome (Aparicio, 2013; Yoshida et al., 2013). Thus, origins can be broadly classified into early and late firing ones, based on their time of activation and, as a consequence, on their ability to fire in the presence of the drug hydroxyurea (HU). Exposure to HU leads to nucleotide depletion and activation of the intra-S phase replication checkpoint with subsequent inhibition of late-origin firing (Zegerman and Diffley, 2010).

The execution of an ordered program of origin activation is a conserved feature of Eukaryotic chromosomes, suggesting that it has an important function in the preservation of the genome (Müller and Nieduszynski, 2012). It remains, however, largely unclear how this program is established. In principle, the task can be achieved by either actively promoting the activity of early origins or by inhibiting that of the late ones, or by a combination of the two (Yoshida et al., 2013). In budding yeast (*Saccharomyces cerevisiae*) and metazoans, early origins appear to selectively benefit from the action of a limited supply of some of the key factors necessary for origin activation, including Cdc45 and the DDK subunit Dbf4 (Collart et al., 2013; Mantiero et al., 2011; Tanaka et al., 2011). It is not known what allows preferential action of these factors at the early origins, and not at the later ones. Clustering of the origins in defined nuclear regions appears to play a role (Duan et al., 2010), as highlighted by a function for the forkhead transcription factors in promoting origin-origin interactions at early replicating regions of the budding yeast genome (Knott et al., 2012).

On the other hand, origin-repressing activities have also been described. An inhibitory function of chromatin on origin action is documented by the role of telomeres (Ferguson and Fangman, 1992), which are late-replicating in yeast, and of heterochromatin-inducing activities such as histone deacetylases (Knott et al., 2009; Vogelauer et al., 2002), in delaying origin firing. A correlation between the nuclear positioning of origins in G1 and their replication timing has been observed (Heun et al., 2001), but artificial tethering of an early origin to the nuclear periphery in yeast (Ebrahimi et al., 2010) or introduction of mutations affecting delocalization of telomeres from the nuclear periphery (Hiraga et al., 2006) were not sufficient to change the replication timing of these

CrossMark

regions, suggesting that the role of nuclear positioning in determining replication timing is likely to be complex and affected by several factors. At telomeres, the Sir3 and Ku proteins have been shown to be required for the late replication of budding yeast subtelomeric regions, suggesting that heterochromatin plays an important role in delaying origin firing at chromosome ends (Stevenson and Gottschling, 1999; Lian et al., 2011; Cosgrove et al., 2002).

The Rif1 protein, originally identified on the basis of its ability to interact with budding yeast telomeric DNA binding protein Rap1 (Hardy et al., 1992), has been found also to be required for the late replication of budding yeast telomeres (Lian et al., 2011). Subsequent work has revealed that, in both fission yeast (*Schizosaccharomyces pombe*) and mammalian cells, Rif1 acts as a general regulator of the origin firing program genome-wide (Hayano et al., 2012; Yamazaki et al., 2012; Cornacchia et al., 2012). The current view is that Rif1 helps establish late-replicating domains and that removal of Rif1 has an indirect knockon effect on early origins. Although the effect of Rif1 on DNA replication is thought to be mediated by its association with chromatin, which in fission yeast only partly relies on its interaction with the telomeric DNA binding factor Taz1 (Tazumi et al., 2012), it remains unknown how Rif1 carries out its repressive action at origins.

RESULTS AND DISCUSSION

Rif1 Interacts with Protein Phosphatase 1

Rif1 has two conserved putative protein phosphatase 1 (PP1) docking motifs (RVxF and SILK type) at its N terminus (Sreesankar et al., 2012). To test whether an interaction with PP1 is important for the role of Rif1 in the control of replication timing, we made an allele of budding yeast RIF1 (*Sc rif1-PP1* allele) carrying two substitutions in each of the conserved motifs (Figure 1A, left; see also Figure S1B). In budding yeast, a single member of the PP1 family is present, encoded by the essential *GLC7* gene, and therefore we set out to investigate whether Rif1 associates with Glc7. Indeed, Myc-tagged Glc7 was able to immunoprecipitate Flag-tagged Rif1 in cell extracts (Figure 1B, lanes 7 and 8), consistent with previous results (Breitkreutz et al., 2010). The amount of Rif1 in the immunoprecipitates was low, possibly as a reflection of low affinity of the interaction, or of differences in relative amounts of the two proteins, or, perhaps more likely, due to competition by other Glc7 binding partners. In any case, importantly, the interaction between the two proteins was not detected in the presence of the *rif1-PP1* mutations (Figure 1B, lanes 9 and 10). We then generated an analogous *rif1-PP1* allele in *S. pombe*, with the same changes in two of the conserved residues in each of the two PP1-interacting motifs (Figure 1A, right; also Figure S1F). In fission yeast, two PP1 family members are present, Dis2 and the less abundant Sds21 (Alvarez-Tabarés et al., 2007). Again, tagged versions of these PP1 proteins were able to immunoprecipitate epitope-tagged Rif1 (Figure 1C, lanes 5 and 11). In this yeast too, the presence of the *rif1-PP1* allele disrupted the interaction (Figure 1C, lanes 6 and 12). Although we, of course, cannot rule out that the interaction between Rif1 and PP1 proteins in either species is indirect, these results suggest that the PP1 docking motifs in Rif1 are functional and promote an interaction with the PP1 phosphatases.

Rif1 Recruits PP1 to Telomeres and to a Late Origin of DNA Replication

The interaction between yeast Rif1 and PP1 raised the possibility that PP1 might be recruited to Rif1-bound chromosomal loci. In budding yeast, chromatin immunoprecipitation (ChIP) revealed robust association of Glc7 with telomeres, which bind Rif1 (Figure 1D). In addition, the binding of Glc7 at both telomeres tested, *VI-R* and *XV-L*, was greatly reduced in the absence of Rif1 and also in the presence of the *rif1-PP1* allele. These data demonstrate that the budding yeast PP1, Glc7, is associated with telomeres at least in part in a Rif1-dependent manner.

To address whether the role of the Rif1/PP1 interaction is restricted to telomeres, we turned to fission yeast where, as in mammals, Rif1 controls replication timing genome-wide. We tested the association of the two *S. pombe* PP1 homologs, Sds21 and Dis2, with a number of chromosomal loci, including a telomere-adjacent region common to the four telomeres of chromosomes I and II, and several origins of DNA replication: the early-firing origins *ori2-326* and *ars2004*, and the late-firing *ars727* and *ori2-4451*, in addition to the centromere of chromosome I (Figure 1E). Because levels of *ars2004* DNA in the immunoprecipitates were very low and independent of *rif1* allelic status (data not shown), we normalized all data to *ars2004*. This analysis revealed strong binding of Sds21 at telomeres that, as observed for Glc7 in *S. cerevisiae*, greatly diminished in the absence of Rif1 or in the presence of Rif1-PP1 (Figure 1E). Interestingly, telomere binding of Dis2 was lower compared to Sds21 (although the latter is less abundant within cells), whereas at *cen1* the situation was reversed and binding of Dis2 was higher compared to Sds21. These results suggest that Sds21 is the primary binding partner of SpRif1 at telomeres, whereas Dis2 might function primarily at centromeres. Importantly, we were able to detect Sds21 binding to one late origin of DNA replication, *ars727*, which previous work has shown to be bound by Rif1 (Figure 1E) (Hayano et al., 2012). Like at telomeres, the binding of Sds21 at *ars727* was strongly affected by mutation of *rif1*. We could not detect binding of Sds21 to the two early origins. However, we also failed to detect Sds21 at the late-firing Rif1-associated origin *ori2-4451*: it is possible that our PCR primers in this case are simply located in an area of low or absent Rif1 binding, which is not homogeneously distributed over late-firing regions (Hayano et al., 2012). Although it remains unclear how pervasively Sds21 (and possibly Dis2) associate with fission yeast origins genome-wide, our results establish that Rif1 recruits PP1 phosphatases to late-replicating telomeric regions in both budding and fission yeast, and to at least one nontelomeric late-firing origin in fission yeast, suggesting that PP1 recruitment is likely to take place at other Rif1-bound origins.

Although Sds21 and Dis2 localize to different cellular and nuclear compartments, there is a degree of overlap in both their localization and function. Dis2, unlike Sds21, is associated with centromeres (Figure 1E), and its absence leads to increased expression and redistribution of Sds21 to these sites, where it is not otherwise normally visualized (Alvarez-Tabarés et al., 2007). Although Sds21 seems to have the primary role in binding

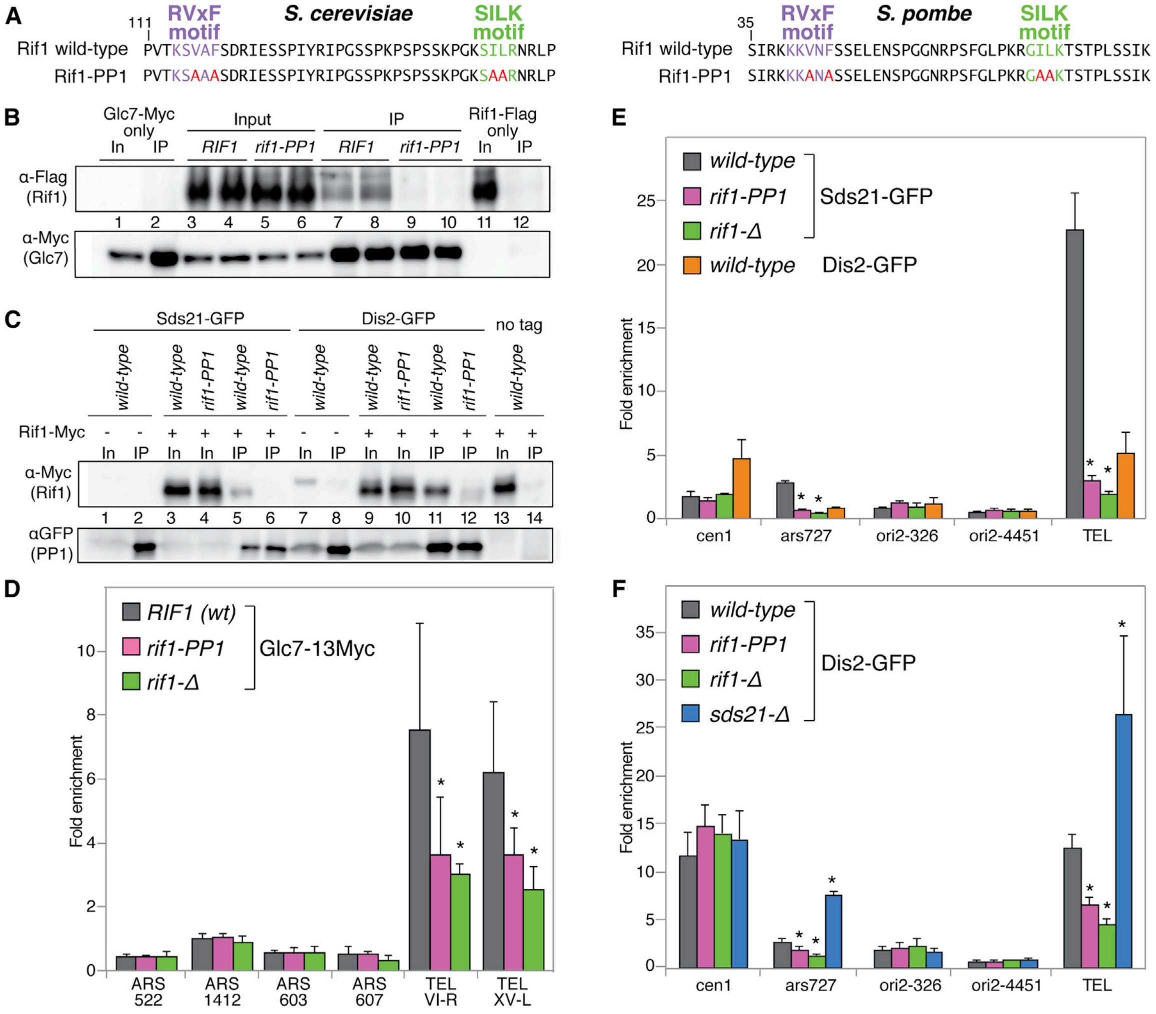

Figure 1. Rif1 Interacts with PP1 and Recruits It to Telomeres

(A) Left: N-terminal sequence of ScRif1 spanning the putative PP1 docking motifs (top), which were mutated in the *rif1-PP1* allele (bottom). Right: N-terminal sequence of SpRif1 spanning the putative PP1 docking motifs (top), mutated in the *rif1-PP1* allele (bottom).

(B) Protein extracts from budding yeast cells of the indicated genotypes were immunoprecipitated with anti-Myc and analyzed by western blotting against Flag (Rif1) and Myc (Glc7).

(C) Protein extracts from fission yeast cells of the indicated genotypes were immunoprecipitated with anti-GFP and analyzed by western blotting against Myc (Rif1) and GFP (Sds21 and Dis2).

(D) ChIP analysis of the association of ScGlc7 with the indicated chromosomal loci in the indicated strains, in exponentially growing asynchronous cultures. Fold enrichment was obtained by normalization against the PDI1 locus. SD values were derived from triplicates, and statistical significance was assessed by determining p values calculated from two-tailed t tests (in all cases, each mutant versus wild-type). *$p < 0.05$.

(E) Association of N-terminally GFP-tagged SpSds21 and SpDis2 from exponentially growing asynchronous cultures at the indicated loci as determined by ChIP and quantified as fold enrichment over the *ars2004* locus. SDs and p values were calculated from four replicates.

(F) ChIP analysis of SpDis2 chromatin binding as in (E); SDs and data are from four replicates.

See Figure S1 for expression levels of mutant alleles.

to telomeres and to *ars727*, the binding of Dis2 to these loci is also dependent on Rif1 (Figure 1F). Interestingly, in both instances, deletion of the *sds21*$^{+}$ gene lead to an increase of about 2.5-fold in the association of Dis2. Thus, similarly to what was previously observed concerning the ability of Sds21 to replace Dis2 at centromeres in its absence, Dis2 increases its association at loci normally occupied by Sds21 in the absence of the latter. The binding of Dis2 at the centromere was instead unaffected either by mutations in Rif1 or Sds21.

The PP1-Interacting Motifs of Rif1 Are Required to Establish the Replication Timing of Telomeric and Nontelomeric Loci

We have previously shown that the timing of association of Pol2 with yeast telomeres reflects their timing of replication and is dependent on the timing of firing of subtelomeric origins (Bianchi and Shore, 2007). In our experiments, Pol2 association with the early origin *ARS607* peaks at 40 min after release from G1 phase, in early S phase, whereas the late origin *ARS1412*

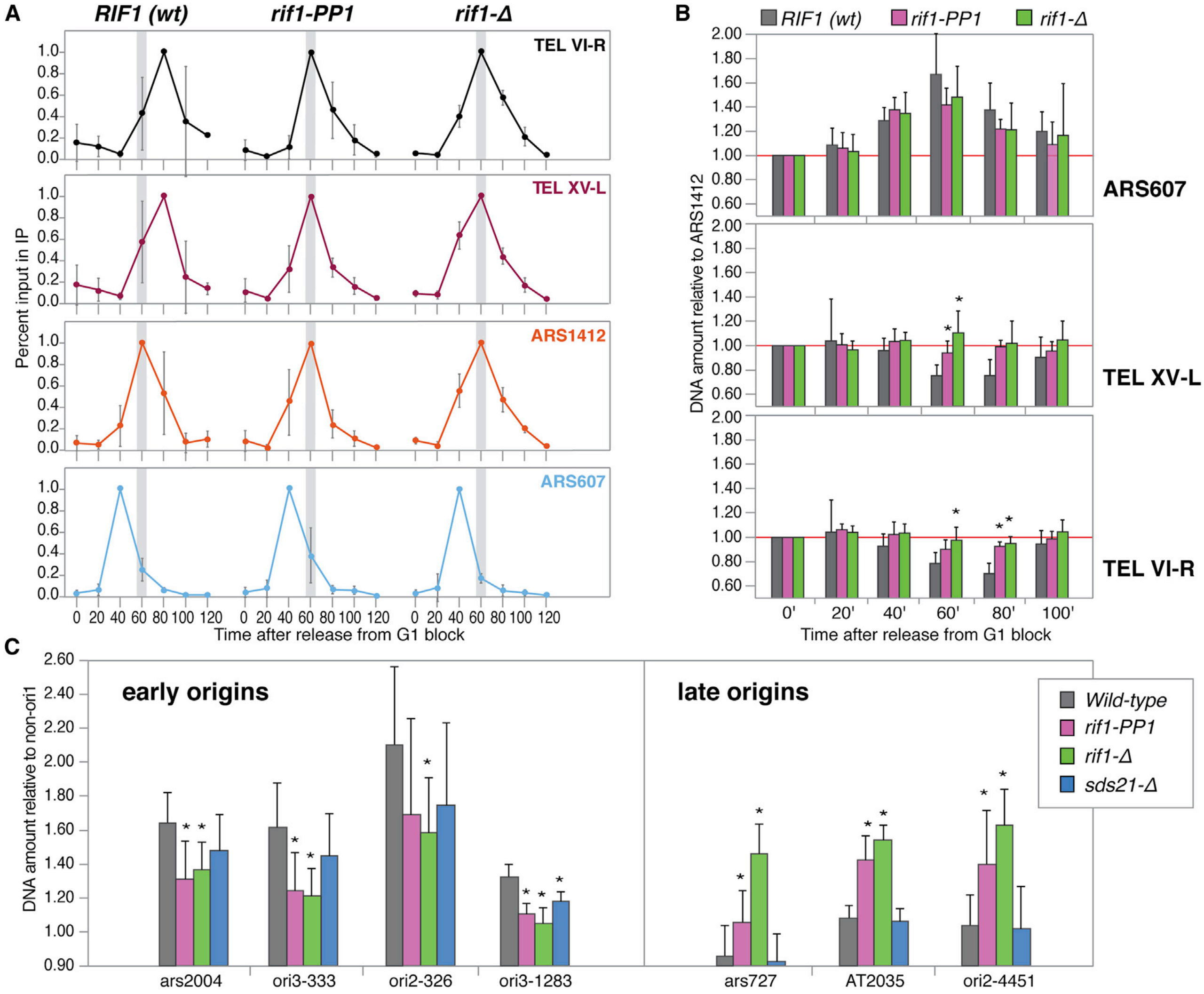

Figure 2. The PP1 Docking Motifs in Rif1 Are Required to Establish the Replication Timing of Budding Yeast Telomeres and Fission Yeast DNA Replication Origins

(A) Analysis of the association of C-terminally Myc-tagged Pol2 with selected telomeres and origins in *RIF1* wild-type, *rif1-PP1*, and *rif1-Δ* budding yeast cells after synchronous release from G1 arrest. ARS607 (blue) and ARS1412 (orange) were used as markers of early and late S phase, respectively. To account for differences in efficiencies in the immunoprecipitations among different experiments, each profile for each amplicon was normalized against its highest peak. The data represent the average of three independent experiments for each strain. The significance of the change in the position of the telomere peak for each rif1 mutant against the wild-type was assessed by applying a Wilcoxon test (one sided; $p < 0.024$).

(B) Analysis of the replication timing of *ARS607* and the *VI-R* and *XV-L* telomeres, in reference to *ARS1412*. DNA amounts for cells after release from G1 arrest were quantified by quantitative PCR (qPCR). For each of the three loci analyzed, normalization was first carried out against the *ARS1412* locus at the same time point, and subsequently against the G1 time point (0 min). A minimum of three experiments were averaged for the analysis. Two-tailed t tests were carried out for significance for each mutant against wild-type at the same time point ($p < 0.05$ is indicated by asterisks). See also Figure S2.

(C) Replication efficiency of early and late origins in fission yeast, in wild-type, *rif1*-Δ, *rif1-PP1*, and *sds21*-Δ strains. Log-phase cultures were arrested in G2 at 36°C using the *cdc25-22* temperature-sensitive allele and then released into 25 mM hydroxyurea for 140 min. Genomic DNA was prepared for the G2 (0 min time point) and late S phase (140 min time point) cells and quantified by qPCR. The ratio of the amount of genomic DNA in late S phase to that in G2 was calculated for each locus. The *non-ori1* locus was used for normalization (Hayano et al., 2012). Two-tailed t test for each mutant against wild-type were performed from at least eight replicates. A p value < 0.05 was deemed significant and is indicated by an asterisk in the graph. SDs are indicated in all panels.

peaks at 60 min (Figure 2A, bottom two panels). Pol2 association with telomeres normally peaks even later in S phase, at 80 min after release (Figure 2A, top two panels, left). Instead, cells carrying the *rif1-PP1* allele displayed a change in telomere Pol2 association, peaking at 60 min, concomitant with binding at *ARS1412* (Figure 2A, top two panels, middle): the extent of the change in Pol2 association is indistinguishable to the one observed in the absence of Rif1 (Figure 2A, top two panels, right), suggesting that the changes in telomere replication timing previously described at budding yeast telomeres in cells lacking Rif1 (Lian et al., 2011) are due to reduced Glc7 telomere binding.

To obtain further evidence that PP1 binding by Rif1 leads to changes in timing of DNA replication at budding yeast telomeres, we quantified the amount of genomic DNA present during S phase progression. Because at the *ARS1412* origin neither the association of Pol2 (Figure 2A) nor the replication timing (Lian et al., 2011) is affected by Rif1, we normalized the data at each individual time point against this locus and against the G1 time point (0 min). In this manner, a locus being replicated before *ARS1412* is predicted to show an increase over the baseline (i.e., to result in values higher than 1) in advance of *ARS1412* replication before returning to the baseline after completion of DNA replication at both loci. Indeed, this is what we observed for the early-firing origin *ARS607* for all strains examined (Figure 2B, top panel). Instead, a locus replicating after *ARS1412*, should show a dip below the baseline coincident with *ARS1412* replication: as expected, this was observed in wild-type cells for both telomeres *VI-R* and *XV-L* (Figure 2B, middle and bottom panels). In contrast, no dip was observed for either telomere in cells lacking *RIF1* or carrying the *rif1-PP1* allele (Figure 2B, middle and bottom panels), indicating that the replication of these telomeres occurs at the same time as *ARS1412* in the presence of the *rif1* mutations, in agreement with the Pol2 ChIP data. These results demonstrate that replication timing at budding yeast telomeres is advanced in cells where the ability of Rif1 to interact with $Glc7^{PP1}$ has been compromised.

Loss of Rif1 can suppress defects in DNA replication in cells that are impaired for DDK function in budding yeast (see below). Although it would seem unlikely that this effect is solely due to the effect of Rif1 at telomeres, we have so far failed to observe binding of ScRif1 (data not shown) or Glc7 (Figure 1D) at origins. However, we have documented mild effects of Rif1 at one late nontelomeric origin, *ARS603*, which would be consistent with a more global role of Rif1 on origin firing (Figure S2).

To further test whether the role of Rif1/PP1 in affecting origin function is widespread in the genome or confined to telomeres, we turned to fission yeast and took advantage of a well-characterized set of early and late origins (Hayano et al., 2012). Cultures were synchronized in the G2 phase at 36°C with a *cdc25-22* temperature-sensitive allele and released into the cell cycle at 25°C in the presence of hydroxyurea, to suppress firing at late origins. DNA amounts after incubation in HU were normalized against a locus (*non-ori*) that is not replicated under these conditions (Hayano et al., 2012), and against the amount at G2 arrest, to provide a measure of the ability of the origins to fire in HU, and therefore of their timing of firing. For the early origins *ars2004*, *ori3-333*, *ori2-326*, *and ori3-1283*, we observed a similar decrease in DNA amounts in *rif1-Δ* and *rif1-PP1* strains, compared to wild-type (Figure 2C, left panel). Analysis of the late origins *ars727*, *AT2035*, and *ori2-4451* instead yielded an increase in DNA amounts in both *rif1-Δ* and *rif1-PP1* strains (Figure 2C, right panel), indicative of a shift to early firing for these late origins. These results demonstrate that, at several loci tested in fission yeast, impairment of the ability of Rif1 to interact with PP1 leads to a loss of control of the timing of origin firing that phenocopies the misregulation observed in the absence of Rif1. We did not observe significant differences in replication efficiencies in the *sds21-Δ* strains compared to wild-type, presumably due to compensatory effects from Dis2.

PP1 Recruitment by Rif1 Affects DDK Action on the MCM Helicase

Loss of Rif1 restores viability of fission yeast cells lacking Hsk1, the catalytic subunit of DDK (Hayano et al., 2012). Similarly, we found that loss of Sc Rif1 partly suppresses the temperature sensitivity of an allele of the $hsk1^{+}$ budding yeast ortholog, *cdc7-1* (Figure 3A). Remarkably, *rif1-PP1* was also able to partly suppress the temperature sensitivity of *cdc7-1*, although to a lesser extent than *rif1-Δ* (Figure 3A). Similarly to budding yeast, fission yeast *rif1-PP1* also restored growth to *hsk1-89* mutants, to an extent comparable to that conferred by *rif1-Δ* (Figure 3B). These results suggest that the Rif1-dependent recruitment of PP1 to replication origins might counteract DDK kinase activity at origins.

To test this idea directly, we assessed Mcm4 phosphorylation in cells carrying mutations in Rif1. Budding yeast Mcm4 has been shown to be a target of multiple phosphorylation events by CDK and DDK (Sheu and Stillman, 2006, 2010; Randell et al., 2010). Although phosphorylation of Mcm4 was not easily apparent in G1-arrested wild-type cells, a supershifted band was readily observable in cells lacking Rif1 (Figure 3C, compare lane 2 with lane 1). This phosphorylation was greatly diminished in the presence of the *cdc7-1* mutation at the permissive temperature and was undetectable at the nonpermissive one (Figure 3C, lanes 5 and 8). These results suggest that phosphorylation of Mcm4 is largely DDK dependent and that this phosphorylation is inhibited by the action of Rif1. Importantly, an increase in phosphorylation of Mcm4 was also observed, to a similar extent, in the presence of the *rif1-PP1* allele (Figure 3C, lanes 3 and 6), suggesting that PP1 activity recruited by Rif1 is responsible for reversal of DDK phosphorylation events.

We did not observe an ability of Rif1 to suppress various budding and fission yeast CDK mutants (Figure S3), but this could be conceivably due to the fact that CDK carries out multiple essential roles other than activation of origin firing. Indeed, the fact that absence of *rif1* suppresses an *hsk1*-null allele in *S. pombe* suggests that reversal of DDK-dependent phosphorylation is not the only function of Rif1/PP1, and that CDK-dependent phosphorylation events might be targeted as well.

Rif1/PP1 Is Affected by Mutations at Putative CDK and DDK Phosphorylation Sites in the Rif1 N Terminus

The PP1 docking domains, in both Sc and Sp Rif1, are embedded in a conserved cluster of putative DDK and CDK sites (Figures 4A, 4D, S1B, and S1F), some of which are known to be phosphorylated (http://www.phosphopep.org). Because precedents exist for inhibition of PP1 binding upon phosphorylation of residues in the proximity of the docking motifs (Kim et al., 2010; Grallert et al., 2013), we considered the possibility that CDK- and DDK-dependent phosphorylation of Rif1 might inhibit PP1 binding, thus helping to enforce the chronological separation of origin firing throughout S phase, as activation of the pre-RC at Rif1-delayed origins would require the levels of the kinases to reach sufficiently high levels for inhibition of PP1 binding. To test this idea, we made mutations in several putative DDK and CDK sites found in the vicinity of the RVxF and SILK motifs in budding yeast Rif1 (Figure 4A). Specifically, we changed nine serine residues to aspartic acid to mimic constitutive

OPEN
ACCESS
CellPress

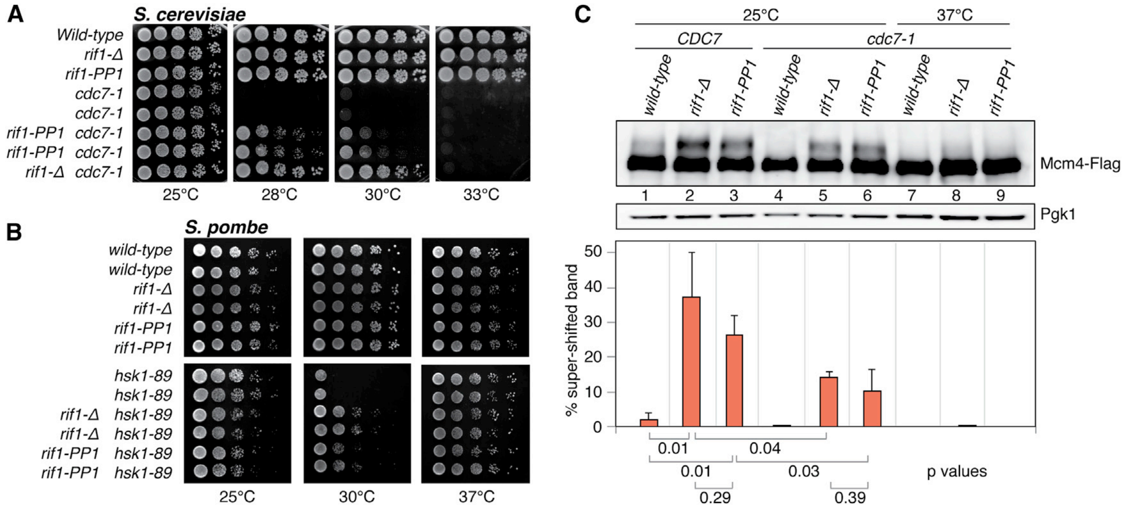

Figure 3. Recruitment of PP1 by Rif1 Counteracts DDK Activity on Mcm4

(A) Suppression of the temperature-sensitivity phenotype of the budding yeast *cdc7-1* allele by *rif1-PP1*. 5-fold serial dilutions of log-phase cultures of budding yeast strains of the indicated genotypes were plated onto YPAD media and incubated at temperatures ranging from 25°C to 33°C. Plates were imaged following 2 day incubations.

(B) Suppression of the temperature sensitivity of the fission yeast *hsk1-89* allele by *rif1-PP1*. 10-fold serial dilutions of log-phase cultures of the indicated genotypes were spotted on rich medium and incubated for 4 days at 25°C or 3 days at 30°C and 37°C (the latter is a permissive temperature for *hsk1-89*).

(C) Analysis of budding yeast Mcm4 phosphorylation. Budding yeast strains bearing Flag-tagged Mcm4 were arrested in the G1 phase with α-factor at 25°C or 37°C for 2 hr, as indicated. Western analysis of protein samples was performed with anti-Flag (top) and anti-Pgk1 (bottom). The phosphorylated fraction of the Mcm4 protein and the total Mcm4 signal were quantified using ImageQuant software and normalized to the loading control (Pgk1), and then the percentage of phosphorylation was calculated. The p values from two-tailed t tests are reported in the graph. At least three replicates were used for the analysis and SDs are indicated.

See also Figure S3.

phosphorylation at these sites, and then we assessed the ability of this mutant (*rif1-9D*) to suppress the temperature-sensitivity phenotype of the *cdc7-1* allele. In agreement with the idea that phosphorylation at these sites might suppress binding of Glc7 to Rif1, we found that the *rif1-9D* phosphomimic allele suppressed growth defects of *cdc7-1* cells to a similar extent as the *rif1-PP1* allele (Figure 4B). Coimmunoprecipitation analysis of this mutant supports this interpretation, because we found that the ability of Glc7 to interact with Rif1 was diminished by the presence of these amino acid substitutions (Figure 4C).

In order to test the potential role of phosphorylation within the N terminus of *S. pombe* Rif1 as well, we made similar phosphomimic substitutions in the protein (Figure 4D, *rif1-12D* allele). As observed in *S. cerevisiae* for *rif1-9D* and *cdc7-1*, the fission yeast allele showed an ability to improve the viability of *hsk1-89* cells (Figure 4E). Strikingly, a second allele where the possibility of CDK and DDK targeting these sites was eliminated by changing serines and threonines to alanines (*rif1-7A*) conferred increased temperature sensitivity to *hsk1-89* cells, as would be expected if this Rif1 protein had enhanced ability to interact with Sds21/Dis2.

We therefore proceeded to monitor the ability of these fission yeast Rif1 proteins to recruit Sds21 to telomeres and to the late origin *ars727*. Consistent with the idea that phosphorylation of the N terminus of Rif1 might downregulate its interaction with PP1, the *rif1-12D* allele displayed impaired recruitment of Sds21 to telomeres and to *ars727* (Figure 4F). The *rif1-7A* allele, on the other hand, led to a strong association of Sds21 at these sites, similar to wild-type. Further analysis will be needed to determine whether the profile of the association of Sds21 with chromatin during the cell cycle is affected in this mutant. Importantly, abrogating the RVxF and SILK domains in the context of the *rif1-7A* allele (*rif1-7APP1* allele) both restored the suppression of *hsk1-89* and impaired the interaction of Sds21 with chromatin, indicating that the synthetic lethality conferred by these alanine substitutions requires the ability of Rif1 to interact with PP1. Taken together, these results suggest that the interaction between Rif1 and PP1 is modulated by kinase activity on the Rif1 N terminus, likely by CDK and DDK.

Conclusions

The key events in the activation of DNA replication are driven by phosphorylation (Labib, 2010). In particular, DDK-dependent phosphorylation of Mcm4 is a key regulatory event in the activation of the pre-RC complex (Tanaka et al., 2011). We show that this event is under control of Rif1-mediated phosphatase action, in agreement with two recent studies in budding yeast (Hiraga et al., 2014; Mattarocci et al., 2014). Our findings suggest that the action of kinases at origins is restricted not only upstream of their action (for example, at recruitment) but also after phosphorylation of their target(s) has occurred. This type of regulation might operate in addition, and in concert, with other modes of origin selection relying on nuclear domain architecture and chromatin accessibility. In this regard Rif1 might have a dual function in chromatin organization and as a recruiter of PP1 at these chromatin domains. Prevention of origin firing by Rif1 would ensure

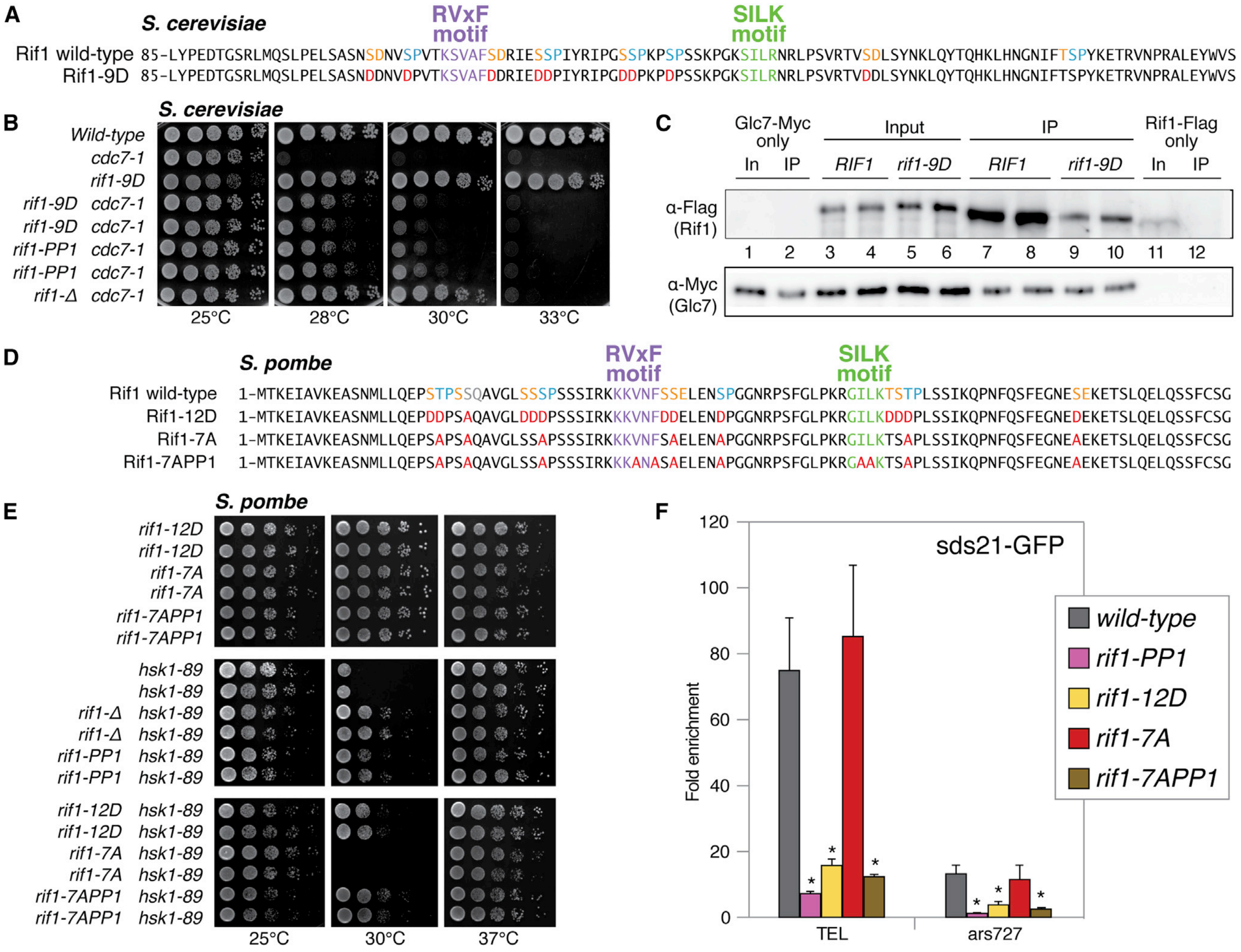

Figure 4. Rif1/PP1 Is Affected by Mutations at Putative CDK and DDK Phosphorylation Sites in the Rif1 N Terminus

(A) N-terminal sequence of ScRif1 spanning the putative PP1 docking motifs (top). The RVxF- and SILK-type motifs are indicated in purple and green, respectively. Potential DDK sites are indicated in orange; putative CDK sites are indicated in blue (top). Phosphomimic changes to aspartic acid present in the *rif1-9D* allele are indicated in red (bottom).

(B) Suppression of the temperature-sensitivity phenotype of the budding yeast *cdc7-1* allele by *rif1-9D*. 5-fold serial dilutions of log-phase cultures of budding yeast strains of the indicated genotypes were plated onto YPAD media and incubated at temperatures ranging from 25°C to 33°C. Plates were imaged following 2 day incubations.

(C) Protein extracts from budding yeast cells of the indicated genotypes were immunoprecipitated with anti-Myc and analyzed by western blotting against Flag (Rif1) and Myc (Glc7).

(D) N-terminal sequence of SpRif1 spanning the putative PP1 docking motifs (top). The RVxF- and SILK-type motifs, and the putative DDK and CDK sites are indicated as in (A). Changes to aspartic acid or alanine present in the *rif1-12D*, *rif1-7A*, and *rif1-7APP1* alleles are indicated in red (bottom).

(E) Suppression of the temperature sensitivity of the fission yeast *hsk1-89* allele by various *rif1* alleles. Ten-fold serial dilutions of log-phase cultures of the indicated genotypes were spotted on rich medium and incubated for 4 days at 25°C or 3 days at 30°C and 37°C.

(F) Association of N-terminally GFP-tagged SpSds21 from exponentially growing asynchronous cultures at the indicated loci as determined by ChIP and quantified as fold enrichment over the *ars2004* locus. SDs and p values for each mutant versus wild-type were calculated from three replicates.

See Figure S1 for expression levels of mutant alleles.

that the limiting factors required for origin activation would be reserved for preferential use at early-firing Rif1-free origins. The delay in firing at many early origins that is observed in the absence of Rif1/PP1 action in fission yeast could be a direct consequence of the scarce availability of limiting factors at early origins due to their increased utilization at misregulated late origins. Release of PP1-dependent inhibition of origin firing by the action of CDK and DDK on Rif1 could provide an additional layer of control on late origin firing and facilitate preferential activation later in the cell cycle at these origins.

Rif1 has a prominent role in orchestrating the replication program in both mouse and human cells (Yamazaki et al., 2012; Cornacchia et al., 2012). Given that mammalian Rif1 has also been shown to bind PP1 (Moorhead et al., 2008), it seems likely that

the role of Rif1-dependent recruitment of PP1 in the control of DNA replication is a conserved feature of eukaryotes. Indeed, PP1 has recently been shown to reverse Cdc7 phosphorylation of MCM in *Xenopus* oocytes (Poh et al., 2014). It will be of interest to address whether PP1 binding might play a role in other processes regulated by Rif1 such as telomerase action and resection of double-strand breaks.

EXPERIMENTAL PROCEDURES

All strains and primers used are listed in Tables S1 and S2, respectively. Procedures for strain handling, construction, and synchronization and for protein extract preparation and analysis as well as for ChIP are given in the Supplemental Experimental Procedures.

SUPPLEMENTAL INFORMATION

Supplemental Information includes Supplemental Experimental Procedures, three figures, and two tables and can be found with this article online at http://dx.doi.org/10.1016/j.celrep.2014.02.019.

ACKNOWLEDGMENTS

We thank Iain Hagan, Kelly Tatchell, Matt Neale, Jorrit Enserink, Hideo Tsubouchi, and Tony Carr for the gift of plasmids and strains and David Shore, Sara Buonomo, and Anne Donaldson for the communication of results prior to publication. We thank Tony Carr for critical reading of the manuscript and Maksym Shyian and David Shore for advice on the Mcm4 gels. This work was supported by a grant from the Medical Research Council to A.B. (G0701428).

Received: November 17, 2013
Revised: January 20, 2014
Accepted: February 14, 2014
Published: March 20, 2014

REFERENCES

Alvarez-Tabarés, I., Grallert, A., Ortiz, J.M., and Hagan, I.M. (2007). Schizosaccharomyces pombe protein phosphatase 1 in mitosis, endocytosis and a partnership with Wsh3/Tea4 to control polarised growth. J. Cell Sci. *120*, 3589–3601.

Aparicio, O.M. (2013). Location, location, location: it's all in the timing for replication origins. Genes Dev. *27*, 117–128.

Bianchi, A., and Shore, D. (2007). Early replication of short telomeres in budding yeast. Cell *128*, 1051–1062.

Breitkreutz, A., Choi, H., Sharom, J.R., Boucher, L., Neduva, V., Larsen, B., Lin, Z.Y., Breitkreutz, B.J., Stark, C., Liu, G., et al. (2010). A global protein kinase and phosphatase interaction network in yeast. Science *328*, 1043–1046.

Collart, C., Allen, G.E., Bradshaw, C.R., Smith, J.C., and Zegerman, P. (2013). Titration of four replication factors is essential for the Xenopus laevis midblastula transition. Science *341*, 893–896.

Cornacchia, D., Dileep, V., Quivy, J.P., Foti, R., Tili, F., Santarella-Mellwig, R., Antony, C., Almouzni, G., Gilbert, D.M., and Buonomo, S.B. (2012). Mouse Rif1 is a key regulator of the replication-timing programme in mammalian cells. EMBO J. *31*, 3678–3690.

Cosgrove, A.J., Nieduszynski, C.A., and Donaldson, A.D. (2002). Ku complex controls the replication time of DNA in telomere regions. Genes Dev. *16*, 2485–2490.

Duan, Z., Andronescu, M., Schutz, K., McIlwain, S., Kim, Y.J., Lee, C., Shendure, J., Fields, S., Blau, C.A., and Noble, W.S. (2010). A three-dimensional model of the yeast genome. Nature *465*, 363–367.

Ebrahimi, H., Robertson, E.D., Taddei, A., Gasser, S.M., Donaldson, A.D., and Hiraga, S. (2010). Early initiation of a replication origin tethered at the nuclear periphery. J. Cell Sci. *123*, 1015–1019.

Ferguson, B.M., and Fangman, W.L. (1992). A position effect on the time of replication origin activation in yeast. Cell *68*, 333–339.

Grallert, A., Chan, K.Y., Alonso-Nuñez, M.L., Madrid, M., Biswas, A., Alvarez-Tabarés, I., Connolly, Y., Tanaka, K., Robertson, A., Ortiz, J.M., et al. (2013). Removal of centrosomal PP1 by NIMA kinase unlocks the MPF feedback loop to promote mitotic commitment in S. pombe. Curr. Biol. *23*, 213–222.

Hardy, C.F., Sussel, L., and Shore, D. (1992). A RAP1-interacting protein involved in transcriptional silencing and telomere length regulation. Genes Dev. *6*, 801–814.

Hayano, M., Kanoh, Y., Matsumoto, S., Renard-Guillet, C., Shirahige, K., and Masai, H. (2012). Rif1 is a global regulator of timing of replication origin firing in fission yeast. Genes Dev. *26*, 137–150.

Heller, R.C., Kang, S., Lam, W.M., Chen, S., Chan, C.S., and Bell, S.P. (2011). Eukaryotic origin-dependent DNA replication in vitro reveals sequential action of DDK and S-CDK kinases. Cell *146*, 80–91.

Heun, P., Laroche, T., Raghuraman, M.K., and Gasser, S.M. (2001). The positioning and dynamics of origins of replication in the budding yeast nucleus. J. Cell Biol. *152*, 385–400.

Hiraga, S., Robertson, E.D., and Donaldson, A.D. (2006). The Ctf18 RFC-like complex positions yeast telomeres but does not specify their replication time. EMBO J. *25*, 1505–1514.

Hiraga, S.-I., Alvino, G.M., Chang, F., Lian, H.-Y., Sridhar, A., Kubota, T., Brewer, B.J., Weinreich, M., Raghuraman, M.K., and Donaldson, A.D. (2014). Rif1 controls DNA replication by directing Protein Phosphatase 1 to reverse Cdc7-mediated phosphorylation of the MCM complex. Genes Dev. *28*, 372–383.

Kim, Y., Holland, A.J., Lan, W., and Cleveland, D.W. (2010). Aurora kinases and protein phosphatase 1 mediate chromosome congression through regulation of CENP-E. Cell *142*, 444–455.

Knott, S.R., Viggiani, C.J., Tavaré, S., and Aparicio, O.M. (2009). Genome-wide replication profiles indicate an expansive role for Rpd3L in regulating replication initiation timing or efficiency, and reveal genomic loci of Rpd3 function in Saccharomyces cerevisiae. Genes Dev. *23*, 1077–1090.

Knott, S.R., Peace, J.M., Ostrow, A.Z., Gan, Y., Rex, A.E., Viggiani, C.J., Tavaré, S., and Aparicio, O.M. (2012). Forkhead transcription factors establish origin timing and long-range clustering in S. cerevisiae. Cell *148*, 99–111.

Labib, K. (2010). How do Cdc7 and cyclin-dependent kinases trigger the initiation of chromosome replication in eukaryotic cells? Genes Dev. *24*, 1208–1219.

Lian, H.Y., Robertson, E.D., Hiraga, S., Alvino, G.M., Collingwood, D., McCune, H.J., Sridhar, A., Brewer, B.J., Raghuraman, M.K., and Donaldson, A.D. (2011). The effect of Ku on telomere replication time is mediated by telomere length but is independent of histone tail acetylation. Mol. Biol. Cell *22*, 1753–1765.

Mantiero, D., Mackenzie, A., Donaldson, A., and Zegerman, P. (2011). Limiting replication initiation factors execute the temporal programme of origin firing in budding yeast. EMBO J. *30*, 4805–4814.

Mattarocci, M., Shyian, M., Lemmens, L., Damay, P., Altintas, D., Shi, T., Bartholomew, C.R., Thomä, N.H., Hardy, C.F.J., and Shore, D. (2014). Rif1 controls DNA replication timing in yeast through the PP1 phosphatase Glc7. Cel Rep *7*. Published online March 27, 2014. http://dx.doi.org/10.1016/j.celrep.2014.03.010.

Moorhead, G.B., Trinkle-Mulcahy, L., Nimick, M., De Wever, V., Campbell, D.G., Gourlay, R., Lam, Y.W., and Lamond, A.I. (2008). Displacement affinity chromatography of protein phosphatase one (PP1) complexes. BMC Biochem. *9*, 28.

Müller, C.A., and Nieduszynski, C.A. (2012). Conservation of replication timing reveals global and local regulation of replication origin activity. Genome Res. *22*, 1953–1962.

Poh, W.T., Chadha, G.S., Gillespie, P.J., Kaldis, P., and Blow, J.J. (2014). Xenopus Cdc7 executes its essential function early in S phase and is counteracted by checkpoint-regulated protein phosphatase 1. Open Biol *4*, 130138.

Randell, J.C., Fan, A., Chan, C., Francis, L.I., Heller, R.C., Galani, K., and Bell, S.P. (2010). Mec1 is one of multiple kinases that prime the Mcm2-7 helicase for phosphorylation by Cdc7. Mol. Cell *40*, 353–363.

Sheu, Y.J., and Stillman, B. (2006). Cdc7-Dbf4 phosphorylates MCM proteins via a docking site-mediated mechanism to promote S phase progression. Mol. Cell *24*, 101–113.

Sheu, Y.J., and Stillman, B. (2010). The Dbf4-Cdc7 kinase promotes S phase by alleviating an inhibitory activity in Mcm4. Nature *463*, 113–117.

Sreesankar, E., Senthilkumar, R., Bharathi, V., Mishra, R.K., and Mishra, K. (2012). Functional diversification of yeast telomere associated protein, Rif1, in higher eukaryotes. BMC Genomics *13*, 255.

Stevenson, J.B., and Gottschling, D.E. (1999). Telomeric chromatin modulates replication timing near chromosome ends. Genes Dev. *13*, 146–151.

Tanaka, S., Umemori, T., Hirai, K., Muramatsu, S., Kamimura, Y., and Araki, H. (2007). CDK-dependent phosphorylation of Sld2 and Sld3 initiates DNA replication in budding yeast. Nature *445*, 328–332.

Tanaka, S., Nakato, R., Katou, Y., Shirahige, K., and Araki, H. (2011). Origin association of Sld3, Sld7, and Cdc45 proteins is a key step for determination of origin-firing timing. Curr. Biol. *21*, 2055–2063.

Tazumi, A., Fukuura, M., Nakato, R., Kishimoto, A., Takenaka, T., Ogawa, S., Song, J.H., Takahashi, T.S., Nakagawa, T., Shirahige, K., and Masukata, H. (2012). Telomere-binding protein Taz1 controls global replication timing through its localization near late replication origins in fission yeast. Genes Dev. *26*, 2050–2062.

Vogelauer, M., Rubbi, L., Lucas, I., Brewer, B.J., and Grunstein, M. (2002). Histone acetylation regulates the time of replication origin firing. Mol. Cell *10*, 1223–1233.

Yamazaki, S., Ishii, A., Kanoh, Y., Oda, M., Nishito, Y., and Masai, H. (2012). Rif1 regulates the replication timing domains on the human genome. EMBO J. *31*, 3667–3677.

Yoshida, K., Poveda, A., and Pasero, P. (2013). Time to be versatile: regulation of the replication timing program in budding yeast. J. Mol. Biol. *425*, 4696–4705.

Zegerman, P., and Diffley, J.F. (2007). Phosphorylation of Sld2 and Sld3 by cyclin-dependent kinases promotes DNA replication in budding yeast. Nature *445*, 281–285.

Zegerman, P., and Diffley, J.F. (2010). Checkpoint-dependent inhibition of DNA replication initiation by Sld3 and Dbf4 phosphorylation. Nature *467*, 474–478.

Cell Reports, Volume *7*

Supplemental Information

Protein Phosphatase 1 Recruitment by Rif1 Regulates DNA Replication Origin Firing by Counteracting DDK Activity

Anoushka Davé, Carol Cooley, Mansi Garg, and Alessandro Bianchi

Protein Phosphatase 1 recruitment by Rif1 regulates DNA replication origin firing by counteracting DDK activity

Anoushka Davé, Carol Cooley, Mansi Garg and Alessandro Bianchi

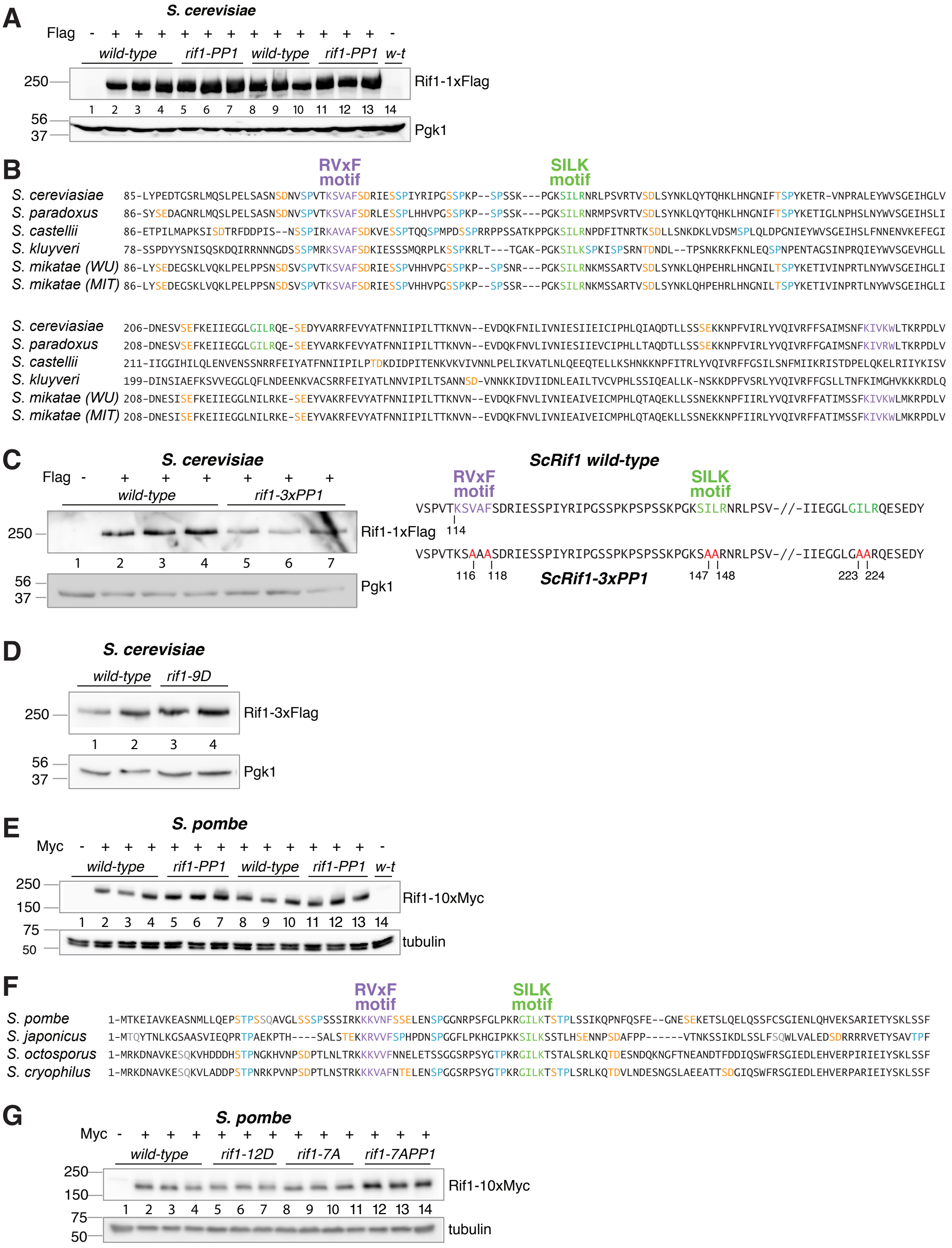

Figure S1. Related to Figures 1 and 4.
Expression levels of mutant Rif1 proteins.

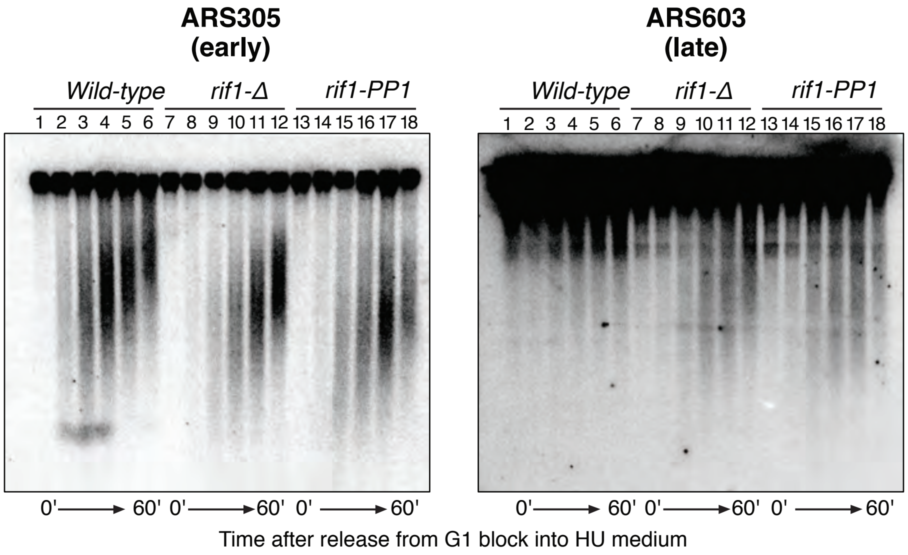

Figure S2. Related to Figure 2.
Loss of Rif1, or of Glc7 binding to Rif1, leads to early activation of late origin *ARS603*.

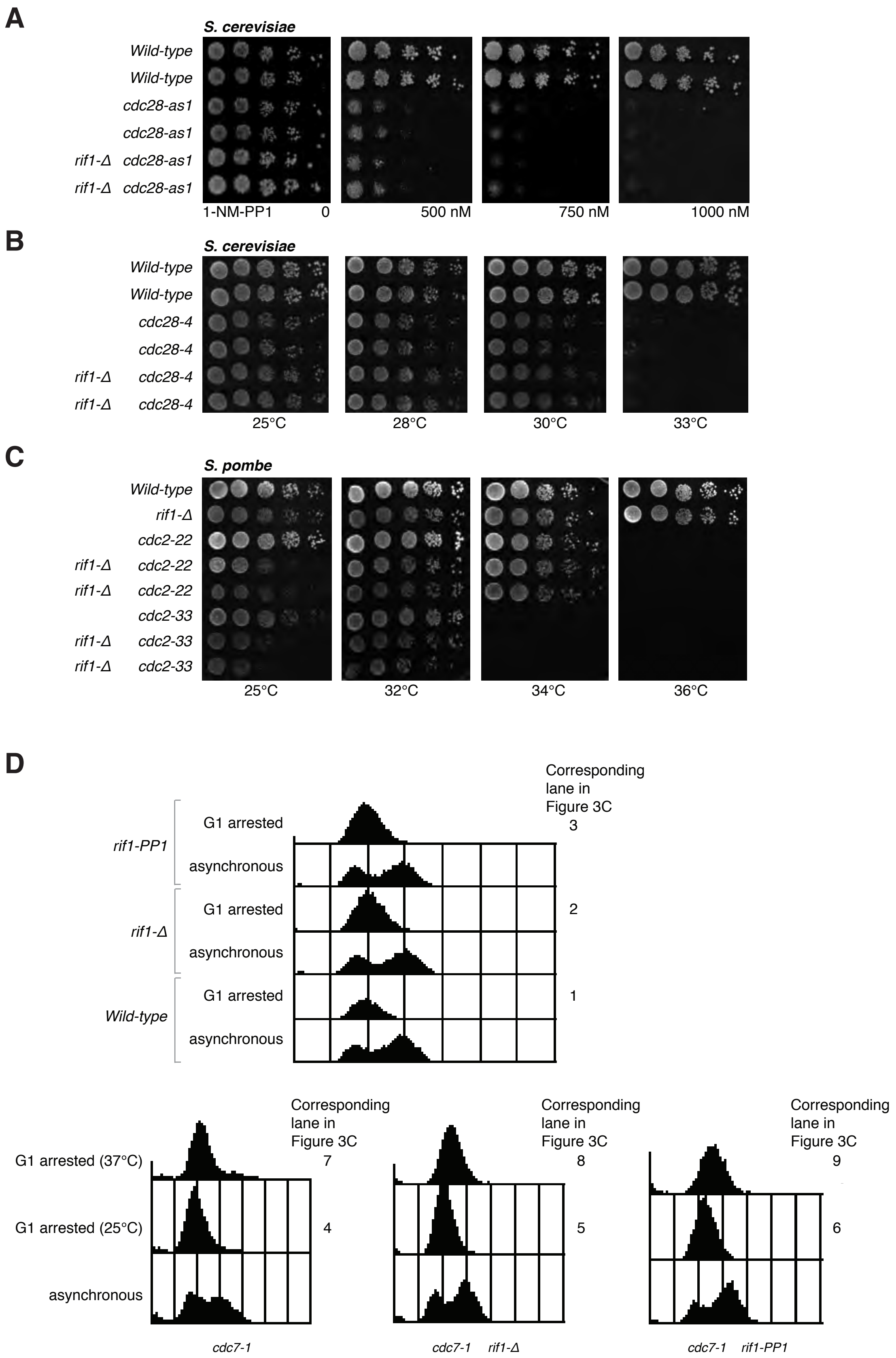

Figure S3. Related to Figure 3.
A-C. Loss of Rif1 does not suppress growth defect of several CDK alleles.
D. FACS analysis of samples from Figure 3C.

Supplemental Figure Legends

Figure S1. Expression levels of mutant Rif1 proteins. Related to Figure 1 and 4.

A. Analysis of protein levels of budding yeast wild-type Rif1 and Rif1-PP1 (referring to Figure 1) tagged at their C-terminus with 1 copy of the Flag epitope, from exponentially growing cultures. The same gel was also blotted for Pgk1, as a loading control. C-terminally tagged versions of the protein product of the *rif1-PP1* allele were found to be present at levels comparable to the wild-type tagged allele. **B.** Alignment of the relevant regions of Rif1 proteins from 5 *Saccharomyces* species (referring to Figure 4A). The RVxF and SILK type motifs are indicated in purple and green, respectively. Putative DDK sites are indicated in orange: these can be 'intrinsic' (SE/SD/TE/TD), or 'phosphorylation generated' where the negative charge C-terminal to the serine or threonine is provided by a prior phosphorylation event (for example by CDK). Finally, putative CDK sites are indicated in blue. **C.** Analysis of protein levels for the budding yeast Rif1 allele bearing changes to alanine at positions 116, 118, 147, 148, 223 and 224 (*rif1-3xPP1*). Although *S. cerevisiae* bears two additional SILK and RVxF type motifs further downstream from the first two (at positions 222 and 316), these putative motifs are embedded within the Ankyrin repeat region of the protein and are not well-conserved within the *Saccharomyces* genus. We found that the *rif1-3xPP1* allele (bearing mutations in the GILR motif at position 222 in addition to those within the first two motifs) was expressed at lower levels, possibly indicative of folding problems. **D.** Analysis of the protein levels of *S. cerevisiae* Rif1-9D, bearing the changes indicated in Figure 4A. **E.** Analysis of protein levels of fission yeast wild-type Rif1 and Rif1-PP1 (referring to Figure 1) tagged at their C-terminus with 10 copies of the Myc epitope, from exponentially growing cultures. The same gel was also blotted for tubulin, as a loading control. C-terminally tagged versions of the protein product of the *rif1-PP1* allele were found to be present at levels comparable to the wild-type tagged allele. **F.** Sequence alignment of the N-terminal region of the *rif1* gene in four *Schizosaccharomyces* species. RVxF (purple) and SILK (green) motifs, and putative DDK (orange) and CDK (blue) sites as above are indicated, in addition to Mec1/Tel1 sites

(grey). **G.** Analysis of the protein levels of *S. pombe* Rif1-12D, Rif1-7A, and Rif1-7APP1, bearing the changes indicated in Figure 4D.

Figure S2. Loss of Rif1, or of Glc7 binding to Rif1, leads to early activation of late origin ARS603. Related to Figure 2.

Analysis of DNA replication intermediates by alkaline agarose gel electrophoresis. Cells of the indicated genotypes from exponentially growing cultures in YPAD at 25°C were arrested in G1 phase of the cell cycle using 0.24 μM alpha-factor. The cells were then washed and released into S-phase in the presence of 200 mM HU, with time points harvested at 0, 20, 30, 40, 50 and 60 mins. Replication intermediates were then separated on alkaline denaturing agarose gels and analyzed by southern blotting using probes for ARS305 (left panel) and ARS603 (right panel). Probes were generated by PCR using oligos DO1787/1788 and DO2498/2499 respectively.

Figure S3. Loss of Rif1 does not suppress growth defect of several CDK alleles. Related to Figure 3.

A. Analysis of the budding yeast *cdc28-as1* (analog-sensitive) allele in the absence of *RIF1.* 5-fold serial dilutions of log-phase cultures of budding yeast strains of the indicated genotypes were plated onto YPAD media with increasing concentrations of the ATP analog 1-NM-PP1. Plates were imaged following 2-day incubations. **B.** Analysis of the suppression of the temperature-sensitivity phenotype of the budding yeast *cdc28-4* allele in the absence of *RIF1.* 5-fold serial dilutions of log-phase cultures of budding yeast strains of the indicated genotypes were plated onto YPAD and incubated at temperatures ranging from 25°C to 33°C. Plates were incubated for two days and then imaged. **C.** Analysis of temperature sensitive alleles of fission yeast *cdc2* combined with *RIF1* deletion. 10-fold serial dilutions of log-phase cultures of the indicated genotypes were spotted on rich medium and incubated for 3 days at temperatures ranging from 25°C to 36°C. **D.** FACS analysis of samples in Figure 3C. Samples were harvested from the same cell cultures analysed by western

blotting in Figure 3C and fixed in 70% ethanol before being stained with Propidium iodide. The distribution of DNA content was then measured using FACS and the profiles aligned using Cell Quest software (Becton Dickinson).

Table S1. List of strains.

Yeast	Strain	Figure	Genotype	Source
S. cerevisiae	YAB1661	1B, lanes 1, 2; 1D; 4C, lanes 1,2	MATα leu2 ura3 his3 GLC7-13myc::kanMX6	Kelly Tatchell
S. cerevisiae	YAB1680	1B, lanes 11, 12; 4C, lanes 11,12	matα-D ade2-D leu2::Gal-HO::LEU2 lys2-D Pol2-13Myc::HIS3 bar1::natMX6 adh4::pAB942 mnt2::LYS2 RIF1-FLAG::KANMX	This Study
S. cerevisiae	YAB1782	1B, lanes 3, 7; 4C, lane 3,7	MATα GLC7-13myc::kanMX6 RIF1-3xFLAG::URA3	This Study
S. cerevisiae	YAB1783	1B, lanes 4, 8; 4C, lane 4,8	MATα GLC7-13myc::kanMX6 RIF1-3xFLAG::URA3	This Study
S. cerevisiae	YAB1784	1B, lanes 5, 9	MATα GLC7-13myc::kanMX6 rif1-PP1-3xFLAG::URA3	This Study
S. cerevisiae	YAB1785	1B, lanes 6, 10	MATα GLC7-13myc::kanMX6 rif1-PP1-3FLAG::URA3	This Study
S. pombe	BAF364	1C lanes 1, 2; 1E; 4F	h- ura4-D18 leu1-32 sds21-EGFPN::ura4	Iain Hagan
S. pombe	BAF554	1C lanes 3, 5	h+ ura4-D18 leu1-32 sds21-EGFPN::ura4 his2- ade-	This Study
S. pombe	BAF558	1C lanes 4, 6	h+ sds21-EGFPN::ura4 rif1-PP1-10xMyc::leu1 ade6-216 ura4-D18 leu1-32	This Study
S. pombe	BAF366	1C lanes 7, 8; 1E; 1F	h- ura4-D18 leu1-32 dis2.NEGFP::ura4	Iain Hagan
S. pombe	BAF556	1C lanes 9, 11	h- ura4-D18 leu1-32 dis2.NEGFP::ura4 rif1-10xMyc::leu1	This Study
S. pombe	BAF561	1C lanes 10, 12	h+ dis2.NEGFP::ura4 rif1-PP1-10xMyc::leu1 ade6-216 his3-D1 ura4-D18 leu1-32	This Study
S. pombe	BAF552	1C lanes 13, 14; S1G, lane2	h+ rif1-10xMyc::leu1 ade6-216 his3-D1 ura4-D18 leu1-32	This Study
S. cerevisiae	YAB1709	1D	MATα GLC7-13myc::kanMX6 rif1-PP1	This Study
S. cerevisiae	YAB1710	1D	MATα GLC7-13myc::kanMX6 rif1-PP1	This Study
S. cerevisiae	YAB1711	1D	MATα GLC7-13myc::kanMX6 rif1-PP1	This Study
S. cerevisiae	YAB1712	1D	MATα GLC7-13myc::kanMX6 rif1::URA3	This Study
S. cerevisiae	YAB1714	1D	MATα GLC7-13myc::kanMX6 rif1::URA3	This Study
S. cerevisiae	YAB1715	1D	MATα GLC7-13myc::kanMX6 rif1::URA3	This Study
S. pombe	BAF365	1E	h+ ura4-D18 leu1-32 sds21-EGFPN::ura4 his2- ade-	Iain Hagan
S. pombe	BAF407	1E; 4F	h+ sds21-EGFPN::ura4 rif1-PP1 ade6-216 ura4-D18 leu1-32	This Study
S. pombe	BAF409	1E	h- sds21-EGFPN::ura4 Rif1::bsd ura4-D18 leu1-32	This Study
S. pombe	BAF410	1E	h- sds21-EGFPN::ura4 Rif1::bsd ade6-704 ura4-D18 leu1-32	This Study
S. pombe	BAF411	1E	h+ sds21-EGFPN::ura4 Rif1::bsd ade6-704 ura4-D18 leu1-32	This Study
S. pombe	BAF408	1F	h+ dis2.NEGFP::ura4 rif1-PP1 ade6-216 his3-D1 ura4-D18 leu1-32	This Study
S. pombe	BAF412	1F	h+ dis2.NEGFP::ura4 Rif1::bsd ade6-704 ura4-D18 leu1-32 nr1	This Study
S. pombe	BAF415	1F	h+ dis2.NEGFP::ura4 Rif1::bsd ade6-704 ura4-D18 leu1-32 nr2	This Study
S. pombe	BAF494	1F	h+ dis2.NEGFP::ura4 sds21::LEU2 ade6-216 his3-D1 ura4-D18 leu1-32	This Study
S. pombe	BAF495	1F	h- dis2.NEGFP::ura4 sds21::LEU2 ade6-216 ura4-D18 leu1-32	This Study
S. pombe	BAF496	1F	h+ dis2.NEGFP::ura4 sds21::LEU2 ura4-D18 leu1-32	This Study
S. pombe	BAF497	1F	h- dis2.NEGFP::ura4 sds21::LEU2 ade6-216 ura4-D18 leu1-32	This Study
S. cerevisiae	YAB1409	2A, left panels; 2B; S1A, lane 1, 14	matα-D ade2-D leu2::Gal-HO::LEU2 lys2-D Pol2-13Myc::HIS3 bar1::natMX6 adh4::pAB942 mnt2::LYS2	This Study
S. cerevisiae	YAB1410	2A, left panels	matα-D ade2-D leu2::Gal-HO::LEU2 lys2-D Pol2-13Myc::HIS3 bar1::natMX6 adh4::pAB704 mnt2::LYS2	This Study
S. cerevisiae	YAB1689	2A, center panels; 2B	matα-D ade2-D leu2::Gal-HO(LEU2) lys2-D Pol2-13Myc::HIS3 bar1::natMX6 adh4::pAB942 mnt2::LYS2 rif1-PP1	This Study
S. cerevisiae	YAB1706	2A, center panels	matα-D ade2-D leu2::Gal-HO(LEU2) lys2-D Pol2-13Myc::HIS3 bar1::natMX6 adh4::pAB704 mnt2::LYS2 rif1-PP1	This Study
S. cerevisiae	YAB1713	2A, center panels; 2B	matα-D ade2-D leu2::Gal-HO(LEU2) lys2-D Pol2-13Myc::HIS3 bar1::natMX6 adh4::pAB942 mnt2::LYS2 rif1-PP1	This Study
S. cerevisiae	YAB1618	2A, right panels; 2B	matα-D ade2-D leu2::Gal-HO(LEU2) lys2-D Pol2-13Myc::HIS3 bar1::natMX6 adh4::pAB942 mnt2::LYS2 rif1::TRP1	This Study
S. pombe	BAF392	2C	h- cdc25-22 cdc20-GFP:KanMX6 ura4-D18 leu1-32 ade6-216	This Study
S. pombe	BAF395	2C	h+ cdc25-22 cdc20-GFP:KanMX6 rif1-PP1 ura4-D18	This Study
S. pombe	BAF396	2C	h- cdc25-22 cdc20-GFP:KanMX6 rif1-PP1 ura4-D18	This Study
S. pombe	BAF401	2C	h+ rif1::bsd cdc25-22 cdc20-GFP:KanMX6 ade6-704 ura4-D18 leu1-32	This Study
S. pombe	BAF463	2C	h+ sds21::LEU2 cdc25-22 cdc20-GFP:KanMX6 ade6-216 his3-D1 ura4-D18 leu1-32	This Study
S. cerevisiae	YAB41	3A	MATα cdc7-1	Lab collection
S. cerevisiae	YAB1739	3A	MATα cdc7-1 rif1-PP1	This Study
S. cerevisiae	YAB1740	3A	MATα cdc7-1 rif1-PP1	This Study
S. cerevisiae	YAB1748	3A	MATα cdc7-1 rif1::TRP1	This Study
S. cerevisiae	YAB0	3A, S1C, lane 1; S2, lanes 1-6 both panels; S3B	MATa	Lab collection
S. cerevisiae	YAB1803	3A; S2, lane 7-13 both panels	MATa rif1::TRP1	This Study
S. cerevisiae	YAB1697	3A; S2, lanes 13–18 both panels	MATa rif1-PP1	This Study
S. pombe	BAF59	3B; S3C	h+ rif1::bsd ade6-704 ura4-D18 leu1-32	This Study
S. pombe	BAF394	3B	h+ rif1-PP1 ade6-216 his3-D1 ura4-D18 leu1-32	This Study
S. pombe	BAF400	3B; 4E	h- hsk1-89:ura4 ura4-D18 leu1-32	Tony Carr
S. pombe	BAF464	3B; 4E	h+ rif1::bsd hsk1-89:ura4 ura4-D18 leu1-32	This Study
S. pombe	BAF484	3B; 4E	h- rif1-PP1 hsk1-89:ura4 ura4-D18 leu1-32	This Study
S. pombe	BAF6	3B; S1E; S1G; S3C	h+ ade6-216 his3-D1 ura4-D18 leu1-32	This Study
S. cerevisiae	YAB1799	3C, lane 1; S3D	MATa mcm4-1xFlag::kanMX	This Study
S. cerevisiae	YAB1805	3C, lane 2; S3D	MATa Rif1::TRP1 Mcm4-1xFlag::kanMX	This Study
S. cerevisiae	YAB1801	3C, lane 3; S3D	MATa rif1-PP1 mcm4-1xFlag::kanMX	This Study
S. cerevisiae	YAB1913	3C, lane 4, 7; S3D	MATa cdc7-1 mcm4-1xFlag::kanMX	This Study

S. cerevisiae	YAB1888	3C, lane 5, 8; S3D	MATa cdc7-1 Rif1::TRP1 Mcm4-1xFlag::kanMX	This Study
S. cerevisiae	YAB1904	3C, lane 6, 9; S3D	MATa cdc7-1 rif1-PP1 mcm4-1xFlag::kanMX	This Study
S. cerevisiae	YAB1902	4B	MATa rif1-9D	This Study
S. cerevisiae	YAB1832	4B	MATα cdc7-1 rif1-9D	This Study
S. cerevisiae	YAB1834	4B	MATα cdc7-1 rif1-9D	This Study
S. cerevisiae	YAB1875	4C	MATα GLC7-13myc::kanMX6 rif1-9D-3xFLAG::URA3	This Study
S. cerevisiae	YAB1877	4C	MATα GLC7-13myc::kanMX6 rif1-9D-3xFLAG::URA3	This Study
S. pombe	BAF548	4E	h+ rif1-12D ade6-216 his3-D1 ura4-D18 leu1-32	This Study
S. pombe	BAF550	4E	h+ rif1-7A ade6-216 his3-D1 ura4-D18 leu1-32	This Study
S. pombe	BAF551	4E	h+ rif1-7APP1 ade6-216 his3-D1 ura4-D18 leu1-32	This Study
S. pombe	BAF604	4E	h- hsk1-89:ura4 rif1-12D ade6-216 ura4-D18 leu1-32	This Study
S. pombe	BAF605	4E	h+ hsk1-89:ura4 rif1-12D his3-D1 ura4-D18 leu1-32	This Study
S. pombe	BAF606	4E	h- hsk1-89:ura4 rif1-7A his3-D1 ura4-D18 leu1-32	This Study
S. pombe	BAF607	4E	h+ hsk1-89:ura4 rif1-7A ade6-216 his3-D1 ura4-D18 leu1-32	This Study
S. pombe	BAF608	4E	h- hsk1-89:ura4 rif1-7APP1 ura4-D18 leu1-32	This Study
S. pombe	BAF609	4E	h+ hsk1-89:ura4 rif1-7APP1 ade6-216 his3-D1 ura4-D18 leu1-32	This Study
S. pombe	BAF621	4F	h+ sds21-EGFPN::ura4 rif1-12D ade6-216 his3-D1 ura4-D18 leu1-32	This Study
S. pombe	BAF622	4F	h- sds21-EGFPN::ura4 rif1-12D ura4-D18 leu1-32	This Study
S. pombe	BAF624	4F	h- sds21-EGFPN::ura4 rif1-7A his3-D1 ura4-D18 leu1-32	This Study
S. pombe	BAF625	4F	h+ sds21-EGFPN::ura4 rif1-7APP1 his3-D1 ura4-D18 leu1-32	This Study
S. pombe	BAF626	4F	h- sds21-EGFPN::ura4 rif1-7APP1 ade6-216 ura4-D18 leu1-32	This Study
S. cerevisiae	YAB1703	S1A, lane 11	matα-D ade2-D leu2::Gal-HO::LEU2 lys2-D Pol2-13Myc::HIS3 bar1::natMX6 adh4::pAB942 mnt2::LYS2 RIF1-1xFLAG::kanMX rif1-PP1	This Study
S. cerevisiae	YAB1704	S1A, lane 12	matα-D ade2-D leu2::Gal-HO::LEU2 lys2-D Pol2-13Myc::HIS3 bar1::natMX6 adh4::pAB942 mnt2::LYS2 RIF1-1xFLAG::kanMX rif1-PP1	This Study
S. cerevisiae	YAB1705	S1A, lane 13	matα-D ade2-D leu2::Gal-HO::LEU2 lys2-D Pol2-13Myc::HIS3 bar1::natMX6 adh4::pAB942 mnt2::LYS2 RIF1-1xFLAG::kanMX rif1-PP1	This Study
S. cerevisiae	YAB1678	S1A, lane 2-4; S1C, lane 2-4	matα-D ade2-D leu2::Gal-HO::LEU2 lys2-D Pol2-13Myc::HIS3 bar1::natMX6 adh4::pAB942 mnt2::LYS2 RIF1-1xFLAG::kanMX	This Study
S. cerevisiae	YAB1700	S1A, lane 5	matα-D ade2-D leu2::Gal-HO::LEU2 lys2-D Pol2-13Myc::HIS3 bar1::natMX6 adh4::pAB942 mnt2::LYS2 RIF1-1xFLAG::kanMX rif1-PP1	This Study
S. cerevisiae	YAB1701	S1A, lane 6	matα-D ade2-D leu2::Gal-HO::LEU2 lys2-D Pol2-13Myc::HIS3 bar1::natMX6 adh4::pAB942 mnt2::LYS2 RIF1-1xFLAG::kanMX rif1-PP1	This Study
S. cerevisiae	YAB1702	S1A, lane 7	matα-D ade2-D leu2::Gal-HO::LEU2 lys2-D Pol2-13Myc::HIS3 bar1::natMX6 adh4::pAB942 mnt2::LYS2 RIF1-1xFLAG::kanMX rif1-PP1	This Study
S. cerevisiae	YAB1679	S1A, lane 8-10	matα-D ade2-D leu2::Gal-HO::LEU2 lys2-D Pol2-13Myc::HIS3 bar1::natMX6 adh4::pAB942 mnt2::LYS2 RIF1-1xFLAG::kanMX	This Study
S. cerevisiae	YAB1681	S1C, lane 5	matα-D ade2-D leu2::Gal-HO::LEU2 lys2-D Pol2-13Myc::HIS3 bar1::natMX6 adh4::pAB942 mnt2::LYS2 RIF1-1xFLAG::kanMX rif1-3xPP1	This Study
S. cerevisiae	YAB1682	S1C, lane 6	matα-D ade2-D leu2::Gal-HO::LEU2 lys2-D Pol2-13Myc::HIS3 bar1::natMX6 adh4::pAB942 mnt2::LYS2 RIF1-1xFLAG::kanMX rif1-3xPP1	This Study
S. cerevisiae	YAB1683	S1C, lane 7	matα-D ade2-D leu2::Gal-HO::LEU2 lys2-D Pol2-13Myc::HIS3 bar1::natMX6 adh4::pAB942 mnt2::LYS2 RIF1-1xFLAG::kanMX rif1-3xPP1	This Study
S. cerevisiae	YAB1843	S1D, lane 1	MATa Rif1-3Flag	This Study
S. cerevisiae	YAB1844	S1D, lane 2	MATa Rif1-3Flag	This Study
S. cerevisiae	YAB1841	S1D, lane 3	MATα Rif1-3Flag, rif1-9D	This Study
S. cerevisiae	YAB1842	S1D, lane 4	MATα Rif1-3Flag, rif1-9D	This Study
S. pombe	BAF486	S1E	h+ rif1-10xMyc::ura4 ade6-216 his3-D1 ura4-D18 leu1-32	This Study
S. pombe	BAF487	S1E	h+ rif1-PP1-10xMyc::ura4 ade6-216 his3-D1 ura4-D18 leu1-32	This Study
S. pombe	BAF488	S1E	h+ rif1-PP1-10xMyc::ura4 ade6-216 his3-D1 ura4-D18 leu1-32	This Study
S. pombe	BAF489	S1E	h+ rif1-PP1-10xMyc::ura4 ade6-216 his3-D1 ura4-D18 leu1-32	This Study
S. pombe	BAF490	S1E	h+ rif1-PP1-10xMyc::ura4 ade6-216 his3-D1 ura4-D18 leu1-32	This Study
S. pombe	BAF491	S1E	h+ rif1-PP1-10xMyc::ura4 ade6-216 his3-D1 ura4-D18 leu1-32	This Study
S. pombe	BAF492	S1E	h+ rif1-PP1-10xMyc::ura4 ade6-216 his3-D1 ura4-D18 leu1-32	This Study
S. pombe	BAF553	S1G, lane 3, 4	h+ rif1-10xMyc::leu1 ade6-216 his3-D1 ura4-D18 leu1-32	This Study
S. pombe	BAF618	S1G, lane 5, 6, 7	h+ rif1-12D-10xMyc::leu1 ade6-216 his3-D1 ura4-D18 leu1-32	This Study
S. pombe	BAF619	S1G, lane 8 ,9, 10	h+ rif1-7A-10xMyc::leu1 ade6-216 his3-D1 ura4-D18 leu1-32	This Study
S. pombe	BAF620	S1G, lane 11, 12, 13	h+ rif1-7APP1-10xMyc::leu1 ade6-216 his3-D1 ura4-D18 leu1-32	This Study
S. cerevisiae	YAB1910	S3A	SK1 MATa ho:LYS2 lys2 ura3 leu2::hisG his3::hisG trp1::hisG	Hideo Tsubouchi
S. cerevisiae	YAB1847	S3A	SK1 MATα ho::LYS2 lys2 ura3 arg4-nsp leu2::hisG cdc28as1	Matt Neale
S. cerevisiae	YAB1900	S3A	SK1 MATα ho::LYS2 lys2 ura3 arg4-nsp leu2::hisG cdc28as1 rif1::TRP1	This Study
S. cerevisiae	YAB1868	S3B	MATa cdc28-4	Jorrit Enserink
S. cerevisiae	YAB1914	S3B	MATa cdc28-4 rif1::TRP1	This Study
S. pombe	BAF591	S3C	h- cdc2-22	Tony Carr
S. pombe	BAF612	S3C	h+ cdc2-22 rif1::bsd ura4-D18 leu1-32	This Study
S. pombe	BAF613	S3C	h- cdc2-22 rif1::bsd ade6-704 ura4-D18	This Study
S. pombe	BAF590	S3C	h- cdc2-33 ade6-704 leu1-32 ura4-D18	Tony Carr
S. pombe	BAF614	S3C	h- cdc2-33 ade6-704 ura4-D18 leu1-32	This Study
S. pombe	BAF615	S3C	h+ cdc2-33 ade6-704 ura4-D18 leu1-32	This Study

NOTES: In the strains bearing the Gal-HO cassette there is no HO site in the genome (the endogenous site at the MAT locus has been deleted) and therefore no HO cleavage is induced, and no effect on cell cycle progress is elicited. pAB942 introduces an ADE2 marker and a stretch of telomeric repeats at the subtelomere of VII-L, with no effects on the other loci and telomeres analyzed. pAB704 (present in strains YAB1410 and YAB1706) is similar to pAB942 but carries an HO site: this plasmid will introduce a DSB at the ADH4 locus, with no consequences on cell cycle progression or replication timing of other loci, as the break is flanked by telomeric arrays.

Table S2. List of oligonucleotides.

Oligo name	Purpose	Name of target locus/template	Oligo sequence
DO1344	QPCR	Sp TEL (subtelomeric)	TATTTCTTTATTCAACTTACCGCACTTC
DO1348	QPCR	Sp TEL (subtelomeric)	CAGTAGTGCAGTGTATTATGATAATTAAAATGG
DO2374	QPCR	Sp non-ori1	TTCAGCGCAAGAACTATTGG
DO2375	QPCR	Sp non-ori1	CACATAACCCGCTAGCTTCC
DO2391	QPCR	Sp 3ori333	AAAATGCCTTGTGCTTTGTG
DO2392	QPCR	Sp 3ori333	GGCCGATCGTCCAGAAATTA
DO2393	QPCR	Sp 3ori1283	GCAGAAGCAGAAGTTCAAAGG
DO2394	QPCR	Sp 3ori1283	AACCAGATTCTTCGGTGCAG
DO2395	QPCR	Sp ars727	CCCCACAAGGTACGAAAAAG
DO2396	QPCR	Sp ars727	TACTCATTTCCCCCACCTCA
DO2401	QPCR	Sp AT2035	TGGTACGTCGAGTGAGACACA
DO2402	QPCR	Sp AT2035	CACTCCGGGGAAGAGGTTAT
DO2406	QPCR	Sp cen1	TCAATTCTGTAAGTTTGCTGTGC
DO2407	QPCR	Sp cen1	AGGAAACGCCATGGAGTACA
DO2449	QPCR	Sp ars2004	GATTGACTCAGTACACACCACACA
DO2451	QPCR	Sp ars2004	GCAATTGTGATGGAATTGGTT
DO2459	QPCR	Sp 2ori326	GGAAATCGAGCAGAGGTCAG
DO2460	QPCR	Sp 2ori326	TTGACGTTGTGCTAAAAGGTG
DO2463	QPCR	Sp 2ori4451	ACGATGTCATTTGGCACTCA
DO2464	QPCR	Sp 2ori4451	AAAAATTTGGAACACTGCTTGT
DO1230	Deletion of Sp rif1	pAB742	AAACTTATTTGTGCAATTTTTTGGTGCGATCGGTCCCATGAAGCTAAAACATTTTTAAAAGCATAACAGCTGATGTTGCGCACTCAACCCTATCTCGG
DO1231	Deletion of Sp rif1	pAB742	GAACCCCATTAATAAGATTGATTTATGACTAAATTGACCCCAATGCCGGTCCGTAATTCCTGTACCCATGAGTTGAATAACTCGAAATTAACCCTCAC
DO2444	genotyping of Sp rif1-PP1	Sp rif1	AACCTTCAACCCCATCATCA
DO2446	genotyping of Sp rif1-PP1/-12D/-7A/-7APP1	Sp rif1	CCTCGGGAAGACTTGGTGTA
DO2502	genotyping of Sp rif1-12D/-7A/-7APP1	Sp rif1	GATCGGTCCCATGAAGCTAA
DO1135	QPCR	Sc TEL VI-R	CCATGACCCAGTCCTCATTT
DO1136	QPCR	Sc TEL VI-R	TGGCAAGGGTAAAAACCAGT
DO1138	QPCR	Sc ARS607	CTTTAGCTGGGTTTATGGGAGGTT
DO2517	QPCR	Sc ARS607	TAATGCACGAGCCGAAACAA
DO1232	QPCR	Sc TEL XV-L	CCTTACCTCCCCACTCGTTAC
DO2518	QPCR	Sc TEL XV-L	ATCGTGGTTCGCTGTGGTAT
DO2519	QPCR	Sc ARS522	AAGCAAATTGCAGAAGGTTATGAA
DO2520	QPCR	Sc ARS522	TTCAAGGCTCTAGCATATGAAACG
DO2208	QPCR	Sc ARS1412	AAGCAAATTGCAGAAGGTTATGAA
DO2209	QPCR	Sc ARS1412	TTCAAGGCTCTAGCATATGAAACG
DO998	QPCR	Sc ARS603	AATCCACCACAAAGCCCTAA
DO999	QPCR	Sc ARS603	CGAGGGTCGAAATCATCATC
DO373	RIF1 C-terminal FLAG tagging	pAB1090	ATTTTATGATGAGGCTCGAATATTACTCAAACAGGGATAATGATATGAATCGGATCCCCGGGTTAATTAA
DO374	RIF1 C-terminal FLAG tagging	pAB1090	ATTGTAATTAATTTATTGCCATTTTGATCTATTCTACATACTAACAATCAGAATTCGAGCTCGTTTAAAC
DO1734	MCM4 C-terminal FLAG tagging	pAB1090	TTGTCCTTGGCGAGGGTGTAAGGAGATCAGTTCGCCTGAATAACCGTGTCGGGGGAGGCGGGGGTGGA
DO1735	MCM4 C-terminal FLAG tagging	pAB1090	TTAGTATTTATTAATTGTTACGCAGGGAATGATTGTAGTAGACAGCATCAGAATTCGAGCTCGTTTAAAC
DO2667	Genotyping of Sc rif1-PP1	Sc RIF1	AGGAAGCAGGCTAATGCAAA
DO2668	Genotyping of Sc rif1-PP1	Sc RIF1	ACTCTAAGGCGCGAGGATTT
DO1787	PCR of ARS305 probe	ARS305	ATCGTGTAAGCTGGGGTGAC
DO1788	PCR of ARS305 probe	ARS305	GGCAAACGTCCAAAGACAAT
DO2498	PCR of ARS603 probe	ARS603	GGTATTGCTGTTTTAAGTGAG
DO2499	PCR of ARS603 probe	ARS603	CATAGATATCGGGTTACTAAAG

Supplemental Experimental Procedures

Strains and plasmids

All budding yeast strains were generated in the W303 background (*MATa ade2-1 his3-11,15 leu2-3,112 trp1-1 ura3-1 can1-100 RAD5*). A complete list of the strains used is reported in Table S1. Standard budding yeast handling and growth conditions were used. Rich medium was YPAD, and drop-out media were made using pre-made mixes from United States Biological.

Fission yeast strains (containing *ade6-M216 ura4-D18 leu1-32 his3-D1* or combinations thereof) were grown in YES rich medium (Moreno et al., 1991) or YNG minimal medium (2% w/v glucose, 30 mM glutamate, 0.17% w/v YNB mix without amino acids ammonium sulphate or thiamine (United States Biological), 0.53% w/v SC dropout mix (United States Biological), 2% w/v agar, pH 6.0. Transformations were performed using a modified version of the protocol described by Bahler (Bahler et al., 1998): cells were incubated with plasmid DNA, carrier DNA and 40% PEG/LiAc/TE solution for 2 hrs at 30°C, before a 10 min heat shock at 42°C; transformations were subsequently plated out directly onto plates without centrifugation.

C-terminal 1xFlag-tagging of ScRif1 and ScMcm4 was obtained with PCR products generated from pFA6a-6xGLY-1xFLAG-kanMX6 (pAB1090) using oligos DO373/DO374 and DO1734/DO1735 respectively. Alternatively, ScRif1 was C-terminally 3xFlag-tagged using a MfeI-linearized plasmid containing the protein's C-terminus cloned in-frame to a triple Flag tag linked by an 8xGlycine linker (pAB1750). Sc *RIF1* gene deletions were obtained using plasmids pAB1511 (TRP1), pAB1655 (URA3), or pAB1749 (ADE2) linearized with SphI. Integration of the Sc *rif1-PP1* allele was obtained by transformation with pAB1654 linearized with SgrAI: colonies were then streaked on 5-FOA and genotyped by PCR with DO2267/DO2268 (314 bp product) followed by restriction enzyme digest with PstI or PvuII (PstI digestion: 314 bp on *RIF1*, 227 + 87 bp on *rif1-PP1*; PvuII digestion: 314 bp on *RIF1*, 146 + 90 + 78 bp on *rif1-PP1*). The *rif1-9D* allele was made in the same way as for *rif1-PP1* allele: SgrAI digested pAB1752 was used for the initial integration followed by FOA pop-out.

Genotyping was done by PCR using the same primers as for *rif1-PP1* (DO2267/DO2268) and digested with AgeI (*RIF1*: 253 + 61 bp, *rif1-9D*: uncut).

To obtain the Sp *rif1-PP1* allele, the same procedure was used using plasmid pAB1664 linearized with BglII. SacI digestion of a 360 bp PCR product made with DO2444/DO2446 on genomic DNA indicated the presence of the allele (237 + 123 bp in *rif1-PP1*; uncut in WT). To obtain the *rif1-12D/-7A/-7APP1* alleles, BglII-linearized plasmids (pAB1741, 1740, 1742 respectively) containing the mutated region and a *ura*+ marker were transformed into yeast. The resultant colonies were counter-selected on 5'-fluoroorotic acid (5'-FOA) to select for recombination at the Sp *rif1* locus. Fission yeast strains with mutations in putative CDK/DDK phosphorylation sites were verified by PCR using DO2502/DO2446 which results in a 462 bp product. DraIII cleaves *rif1-7A* and *rif1-7APP1* but not *rif1-12D* PCR products to give 260 + 202 bp fragments, while ApoI cleaves *rif1-7A* and *rif1-12D* to give 176, 175 and 111bp fragments, and *rif1-7APP1* to give 287 + 175 bp fragments. All Rif1 mutant alleles were generated by gene synthesis (Eurofins). The C-terminus of Sp Rif1 was tagged with 10xMyc epitopes using pAB1462 or pAB1744 linearized with MfeI. Sp *rif1*+ was deleted using a PCR product made with oligonucleotides DO1230/DO1231 on using pSVEM-bsd (pAB742) as template (Erler et al., 2006). Fission yeast strains with deletions of *sds21*+ or *dis2+,* or with N-terminal EGFP-tags of the same genes expressed from their chromosomal locus were kindly gifted by Iain Hagan. The *hsk1-89* strain was gratefully received from Tony Carr.

Immunoprecipitation of ScRif1

Cultures of exponentially growing budding yeast cells (100 ml of 1 x 10^7 cells/ml) were lysed in 15 mM Hepes pH 7.6, 150 mM NaCl, 0.5 % NP-40 with three 20 s pulses in a Beadbeater. The lysate was clarified by centrifugation and the supernatant was incubated first with anti-myc 9E10 monoclonal antibody for 2 hours at 4°C and then with Protein G Dynabeads for 1 hour at 4°C. Bound proteins (eluted by boiling in Laemmli buffer) and input samples were separated on a 8% (top panel) and 10% (bottom panel) SDS gel and Western blotting was performed using anti-Flag (M2, Sigma) or anti-Myc antibody (homemade 9E10).

Immunoprecipitation of SpRif1

Whole cell extracts from $2x10^8$ fission yeast were prepared by resuspending in 500 µl of chilled lysis buffer (50 mM NaCl, 50 mM Tris pH 7.5, 10% glycerol, 4 mM B-mercaptoethanol, 1 mM EDTA) containing 1 complete protease inhibitor tablet (Roche) per 7 ml of buffer. Following lysis in a beadbeater for 3 min, 1 µl Benzonase Nuclease (Novazyme) and 50 µl 10% NP40 were added to the lysate and incubated for 30 min on ice. After centrifugation for 10 min, 30 µl of the cleared lysate was boiled with 10 µl 4x Laemmli buffer and kept aside as Input. Protein G Dynabeads (Invitrogen) were blocked for 30 min in lysis buffer + 5% BSA. 25 µl of the beads were used to pre-clear the remaining lysate for 30 min at 4°C on a rotating wheel. 1 µl rabbit anti-GFP antibody (Invitrogen) was added to the supernatant obtained after magnetic separation of the beads. The samples were then incubated with rotation at 4°C for 1 hr. 25 µl of Dynabeads were added before further incubation at 4°C for 2 hrs. Following magnetic separation and removal of the supernatant, the beads were resuspended in 40 µl 1x Laemmli buffer and boiled for 5 min.

Synchronization of budding yeast cultures

Budding yeast cells were grown in 100 ml overnight cultures in the appropriate drop-out SC medium containing 4% raffinose. The cultures were then diluted into 300 ml of YPA 4% raffinose and grown for 2 hours with 0.025 µM α-factor to arrest the cells in G1 phase of the cell cycle. Cells at a density of 1×10^7 cells/ml were then switched to YPA 4% galactose for 4 hours at 30°C, while maintaining the arrest with 0.025 µM α-factor. Cells were released into S-phase by washing twice with water and switching the cells to YPA+GAL containing 0.125 mg/ml pronase at 18°C.

Quantification of budding and fission yeast DNA

Fission yeast strains containing the *cdc25-22* allele were grown to mid-log-phase at 25°C, arrested in G2 for 3h at 36°C and subsequently released into medium containing 25 mM HU at 25°C for 140 min. Samples were collected for G2-arrested cells and S-phase-arrested cells (140 min in HU) by cross-linking cells in 1% formaldehyde.

Budding yeast strains were synchronized as described above and samples collected after crosslinking in 1% formaldehyde.

For both yeasts, cell were lysed on a BeadBeater and centrifuged. Pellets were then resuspended in 500 µl ChIP Lysis buffer (50 mM HEPES pH 7.5,140 mM NaCl, 1 mM EDTA, 1% Igepal CA-630, 0.1% sodium deoxycholate) and sonicated 15 x 30 sec on high in a Diagenode Bioruptor. 10 µl of the supernatant collected after centrifugation was added to 110 µl TES (20 mM Tris-Cl pH7.5, 1 mM EDTA, 1% SDS) and de-crosslinked overnight at 65°C. DNAs were purified using the Qiagen PCR purification kit.

ChIP

ChIP was performed as described previously (Bianchi and Shore, 2007). Briefly, after cross-linking in 1% formaldehyde, cells were lysed and sonicated to achieve DNA fragments <500 bp. Immunoprecipitations were carried out with anti-Myc 9E10 (supernatant from a 9E10 hybridoma cell-line) or anti-GFP (Invitrogen) and ProteinG Dynabeads (Invitrogen) against N- or C-terminally tagged proteins, as indicated. Both an aliquot of sonicated cleared extract (input) and the immunoprecipitated material were de-cross-linked in TE plus 1% SDS at 65°C overnight. Quantitation of immunoprecipitated DNA was obtained by real-time PCR using SYBR Green detection on a Roche Light Cycler 480 II instrument and expressed either as percent of starting (input) material or as fold-enrichment over a control locus (Pfaffl, 2001). Primers used are listed in Table S2.

Protein analysis by Western blotting

Protein extracts were made using asynchronous cultures by lysing cells using 0.5 mm glass beads and 200 µl 20% TCA in a Bead-Beater. Next, lysates were added to 400 µl 5% TCA. Cells were then pelleted and resuspended in 200 µl Laemmli sample buffer (250 mM TrisCl pH7.5, 2% SDS, 5% Glycerol, 0.1% BromoPhenol Blue, 2.5% β-mercaptoethanol) and boiled at 95°C for 5min. The samples were then separated on SDS-PAGE gel of required percentage and western blotting was

performed. The proteins were visualized by treating the membrane with ECL and imaged using a LAS 4000 instrument (GE).

Analysis of Mcm4 phosphorylation

Budding yeast cells were grown in 5 ml overnight cultures in YPAD at 25°C (wild-type*, rif1-Δ, rif1-PP1, cdc7-1, cdc7-1 rif1-Δ, cdc7-1 rif1-PP1*). The cultures were then diluted to 0.4 x 10^7 cells/ml in 10 ml YPAD and were grown at 25°C for one cell cycle to obtain a log phase culture. 2.4 µM α-factor was added to arrest the cells in G1 phase of the cell cycle at either 25°C or 37°C for 1 hour. After 1 hour the cells were pelleted and resuspended in fresh 10 ml YPAD with 2.4 µM α-factor and incubated for another hour at the respective temperatures. After a total of 2 hours the cells were harvested and were resuspended in 600 µl 100%TCA and were kept on ice for 10 minutes. The cells were pelleted by centrifuging at 3000 rpm for 2 minutes, followed by two acetone washes. The pellets were then dried under vacuum and were resuspended in 100 µl Urea buffer (50 mM Tris-Cl pH 7.5, 5 mM EDTA, 6 M Urea, 1% SDS). 200 µl 0.5 mm glass beads were added to the tubes and the cells were lysed in a bead beater 5 times for 45 seconds with 1 min on ice in between. The extracts were incubated at 65°C for 10 minutes and centrifuged at 14000 rpm for 10 minutes before the addition of 200 µl of 2x Laemmli buffer. Samples were boiled for 5 min and separated on 6% SDS-PAGE gel.

Alkaline gels

DNA replication intermediates were analyzed using alkaline agarose gel electrophoresis. Briefly, DNA was prepared by lysing 1 x 10^8 cells using zymolyase 100T (USB) extraction method and the DNA was then separated on a 1% denaturing alkaline gel (50 mN NaOH, 1 mM EDTA). Southern blotting was then performed and the DNA was probed with ^{32}P-labelled ARS305 probe and ARS603 probe. Probes were generated by PCR using oligos DO1787/1788 and DODO2498/DO2499.

FACS analysis

Cells were fixed in 70% ethanol and treated with 200 µg/ml RNAse. Cells were then stained

with 10 µg/ml Propidium Iodide (PI) before being analyzed in 50 mM Na citrate 10 µg/ml PI on a Becton Dickinson FACScalibur flow cytometer with CellQuest software.

Supplemental References

Bahler, J., Wu, J.Q., Longtine, M.S., Shah, N.G., McKenzie, A.,.3., Steever, A.B., Wach, A., Philippsen, P., and Pringle, J.R. (1998). Heterologous modules for efficient and versatile PCR-based gene targeting in Schizosaccharomyces pombe. Yeast 14, 943-951.

Bianchi, A., and Shore, D. (2007). Early replication of short telomeres in budding yeast. Cell 128, 1051-1062.

Erler, A., Maresca, M., Fu, J., and Stewart, A.F. (2006). Recombineering reagents for improved inducible expression and selection marker re-use in Schizosaccharomyces pombe. Yeast 23, 813-823.

Moreno, S., Klar, A., and Nurse, P. (1991). Molecular genetic analysis of fission yeast Schizosaccharomyces pombe. Methods Enzymol 194, 795-823.

Pfaffl, M.W. (2001). A new mathematical model for relative quantification in real-time RT-PCR. Nucleic Acids Res 29, e45.
